# Cooperative Transmembrane Penetration of Nanoparticles

**DOI:** 10.1038/srep10525

**Published:** 2015-05-27

**Authors:** Haizhen Zhang, Qiuju Ji, Changjin Huang, Sulin Zhang, Bing Yuan, Kai Yang, Yu-qiang Ma

**Affiliations:** 1 Center for Soft Condensed Matter Physics and Interdisciplinary Research, Soochow University, Suzhou, 215006, China; 2 Department of Engineering Science and Mechanics, The Pennsylvania State University, University Park, Pennsylvania 16802, United States; 3 Collaborative Innovation Center of Advanced Microstructures and Department of Physics, Nanjing University, Nanjing, 210093, China

## Abstract

Physical penetration of lipid bilayer membranes presents an alternative pathway for cellular delivery of nanoparticles (NPs) besides endocytosis. NPs delivered through this pathway could reach the cytoplasm, thereby opening the possibility of organelle-specific targeting. Herein we perform dissipative particle dynamics simulations to elucidate the transmembrane penetration mechanisms of multiple NPs. Our simulations demonstrate that NPs’ translocation proceeds in a cooperative manner, where the interplay of the quantity and surface chemistry of the NPs regulates the translocation efficiency. For NPs with hydrophilic surfaces, the increase of particle quantity facilitates penetration, while for NPs with partly or totally hydrophobic surfaces, the opposite highly possibly holds. Moreover, a set of interesting cooperative ways, such as aggregation, aggregation-dispersion, and aggregation-dispersion-reaggregation of the NPs, are observed during the penetration process. We find that the penetration behaviors of multiple NPs are mostly dominated by the changes of the NP-membrane force components in the membrane plane direction, in addition to that in the penetration direction, suggesting a different interaction mechanism between the multiple NPs and the membrane compared with the one-NP case. These results provide a fundamental understanding in the underlying mechanisms of cooperative penetration of NPs, and shed light on the NP-based drug and gene delivery.

Owing to the excellent physicochemical properties and small size, nanoparticles (NPs) are considered to be one of the promising candidates as drug and gene delivery vectors, intracellular biomarkers and probes, and so on^1^,^2^,^3^. The premise to achieve these biomedical applications is the delivery of NPs into cells with a high efficiency. Endocytosis has been long argued as an effective delivery pathway of NPs^4^,^5^, in which the NPs are wrapped by the cell membrane and then pinch off to cell interior. However, upon internalization, the NPs are often trapped in certain sites along the endocytic routes, such as endosomes and lysosomes^6^,^7^,^8^. Passive transmembrane penetration presents an alternative pathway for cellular delivery of NPs^9^,^10^,^11^,^12^,^13^. It has been observed that penetrated NPs are localized in the cytoplasm, which offers a possibility for organelle-specific targeting^6^,^8^,^14^. Therefore, a fundamental understanding of transmembrane penetration of NPs is critically important to the rational design of NPs with enhanced cellular targeting efficiency.

Externally applied forces and fields may cause membrane pore opening, thereby facilitating direct penetration of NPs. For example, using the microinjection method, NPs can be mechanically delivered into living cells^6^,^15^. Considering the submicron scale self-healing ability of lipid membranes^9^,^16^, nanoneedles can deliver cargos with a high spatial precision while with minimal physical damage to the membrane^8^,^17^. Furthermore, applied electrical field can not only open pores on the cell membrane, but also exert electrostatic forces onto the charged NPs, both of which drive the translocation of NPs^6^,^18^.

The surface chemical properties of NPs strongly affect the interactions between the NPs and the cell membranes^18^,^19^,^20^,^21^,^22^,^23^, thereby modulating the transmembrane penetration of the NPs. NPs with surface patterned chemicals were found to have an astounding ability to pierce the cell membrane^24^,^25^,^26^. Verma *et al.* observed that “striped” NPs could easily pass through the cell membrane^24^, where the entry mechanism was speculated to be involved with the sophisticated interactions between surface composition of NPs and lipid bilayer^27^,^28^. Moreover, the coupling between the surface chemistry and particle geometry makes the interactions of some NPs with the cell membrane very complicated^29^,^30^,^31^,^32^,^33^,^34^,^35^. For example, it is found that the spontaneous insertion of a 2-dimensional graphene sheet, although its surface is hydrophobic, into the membrane is always initiated at sharp corners of the edge to avoid the high energy barrier^33^. Similarly, Zhou and his coworkers found that a swing motion of graphene above the membrane could facilitate its insertion^32^. Surprisingly, due to the unique geometric structure, the lipid extraction from the bilayer by graphene occurs once it pierces into the membrane^32^. However, it is shown that NPs often agglomerate together in the extracellular environment^36^,^37^, meaning that a patch of cell membrane may always “see” multiple NPs simultaneously. Such an agglomeration behavior of NPs, as shown by the simulations of Li *et al.*^13^, may cause the dramatic shape change of a lipid vesicle and even the rupture of the membrane, implying the potential biomedical application^13^. However, how these concentrated NPs work together to affect their interaction with cells and realize the entry into cells, which is although an issue of common concern^38^,^39^,^40^,^41^,^42^,^43^, is still largely unknown^37^.

Herein, we elucidate the cooperative transmembrane penetration mechanisms of multiple NPs using the dissipative particle dynamics (DPD) simulations. We find that the NPs translocate into/through a lipid membrane in a cooperative way, which is further regulated by the interplay of the two key factors, i.e., the quantity and surface chemistry of the NPs. Importantly, the simulations demonstrate that the NP-membrane interaction mechanism in the multiple-NP case is totally different from that in the one-NP case, which fundamentally changes the penetration behavior of multiple NPs. Our findings provide a deep understanding of the collective behavior of the NPs and their interactions with cell membranes, and shed light on the rational design of NP-based diagnostic and therapeutic agents.

## Results

**Figure 1:**
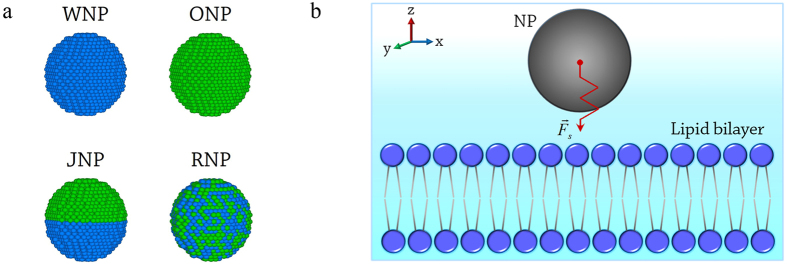
Sketches of NPs and the penetration behavior. (**a**), NPs with different surface chemical properties. Each NP is spherical in shape and its diameter is 4.8 nm. It has been proved by experiments that this particle size is very helpful for the membrane penetration^12^. Beads in blue are hydrophilic, while beads in green are hydrophobic. (**b**), Schematic showing the penetration of the NP into the lipid membrane. A moving spring force 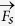 acting on the center-of-mass of a NP to guide the penetration.

Four types of spherical NPs with different surface chemical properties are used in our simulations (Fig. 1a): the WNP with hydrophilic surface, the ONP with hydrophobic surface, the JNP whose surface is half-hydrophilic and half-hydrophobic, and the RNP whose surface is randomly coated with the same ratio of hydrophilic to hydrophobic beads (50% ~ 50%). The dissipative particle dynamics (DPD), which has been extensively used in studies of the nanomaterial-membrane interactions^9^,^20^,^33^,^41^, is applied to simulate the translocation of NPs into/through a lipid membrane. To perform such a biophysical penetration test, a moving spring force (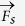) characterized by velocity 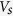 is added on the center-of-mass of the NP (Fig. 1b), as detailed in Methods.

### Penetration of a single NP

**Figure 2:**
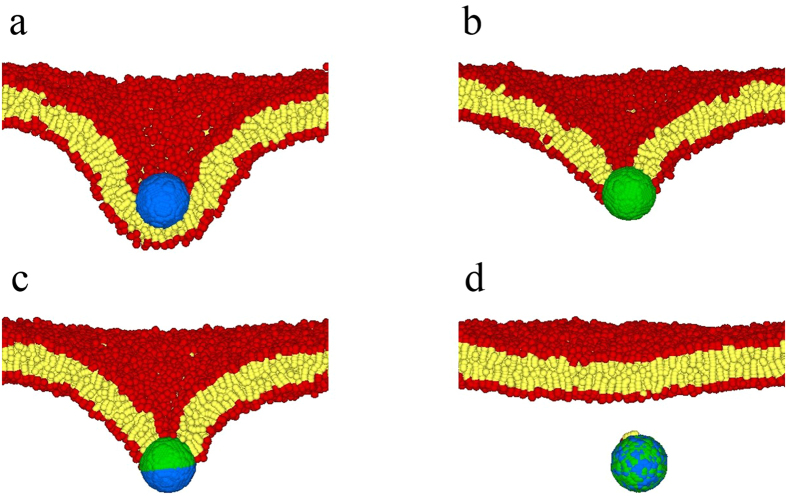
Representative translocation behavior of one single NP . (**a**), The one-WNP case. The WNP is still on the membrane surface at the end of the simulations. (**b**), The one-ONP case. The ONP is inside the membrane. (**c**), The one-JNP case. The JNP is trapped in the membrane. (**d**), The one-RNP case. The RNP completely penetrates through the membrane. The lipid molecules are coarse-grained by connected beads, where beads in red represent lipid heads, while beads in yellow stand for lipid tails. 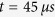, 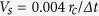.

To better understand the membrane penetration of multiple NPs, we first examine the translocation process of a single NP. As shown in Fig. 2 (more trajectories are shown in Supplementary Fig.S1), under the same simulation conditions, the NP-membrane interaction states at the end of the simulations (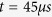) are completely different: the WNP remains on the membrane surface, although the membrane is deformed strongly; the JNP and ONP are trapped in the membrane; whereas the RNP successfully penetrates through the membrane.

During the translocation process, the NP interacts with the hydrophilic outer layer (lipid heads) and hydrophobic inner layer (lipid tails) of the membrane in a consecutive manner. The particle surface chemistry has a rather strong impact on the translocation. By force analysis, it is found that the resistance force acting on the WNP during the translocation is the largest, but that acting on the RNP is the smallest (Supplementary Fig. S2). For the WNP, its resistance force, which is mostly originated from the strong repulsion between the hydrophilic surface of WNP and the hydrophobic inner layer of the bilayer (Supplementary Fig.S3a) and even its interaction with the surrounding water molecules^44^,^45^, always increases with the proceeding of particle translocation (Supplementary Fig. S2). It is therefore difficult for the WNP to enter the membrane interior (Fig. 2). Opposite to the WNP, the ONP, JNP and RNP can easily or even spontaneously translocate into the membrane, as suggested by the sharp drop to zero or even a negative value of their resistance forces after the initial contact of these NPs with the membrane. This point is also confirmed by the previous simulation observations^13^. In comparison with the one-WNP case, the ease of these NPs’ insertion is caused by hydrophobic attraction between the NP surface (hydrophobic part) and the lipid tails, as suggested by the free energy calculation presented by Li and Gao *et al.*^13^,^26^ However, such an attraction would turn to a resistance for the further translocation of these NPs inside the membrane and impede the full penetration (Supplementary Fig. S3). Thus the resistance forces of these NPs increase again after the entry of NPs, and then reach a maximum when they are near the membrane center (Supplementary Fig. S2). As a result, the ONP and JNP are still trapped in the membrane at the end of the simulations (Fig. 2). For the RNP, its random surface chemical pattern cancels the hydrophobic attraction to some extent, and thus reduces its resistance force inside the membrane (Supplementary Fig. S2). Thus the RNP possibly keeps going with the help of the external pulling force till fully passes through the membrane under the similar simulation conditions (Fig. 2). Additionally, the change in the applied velocity 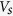 also affects the translocation of NP, but the interaction state is still dependent on the NP’s type (Supplementary Fig. S4). Therefore, all these results demonstrate that the particle surface chemistry indeed modulates a single NP’s penetration behavior.

### Penetration of multiple NPs

**Figure 3:**
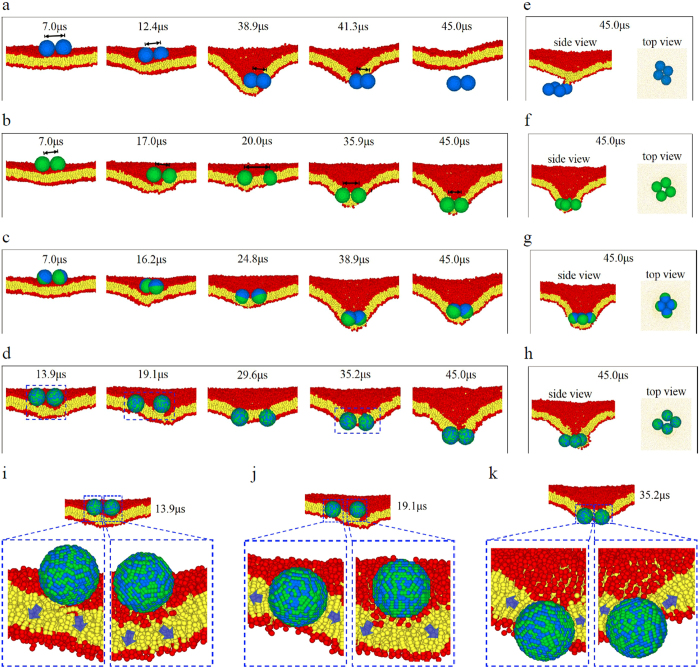
Representative translocation behavior of multiple NPs. (**a-d**), The two-NP cases: **a**, WNPs; **b**, ONPs; **c**, JNPs; **d**, RNPs. The black arrows in figure **a** and **b** are used to demonstrate the change of the inter-NP distance. (**e-h**), The four-NP cases: (**e**), WNPs; (**f**), ONPs; (**g**), JNPs; (**h**), RNPs. The membranes in the top-view figures are displayed semi-transparently. (**i-k**), The magnified images of the local deformations of the membrane near the two RNPs at different stages (labeled by the blue dashed box in figure (**d**). The blue arrows in figure **i-k** show the possible deformation direction of lipid molecules in the membrane. The initial distance between the nearest-neighbor NPs is 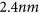, and 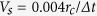.

We next investigate the penetration of multiple NPs, focusing on the effect of NP quantity. Interestingly, it is found that when multiple NPs simultaneously interact with the membrane, the translocation behavior of multiple NPs may be distinctly different from that of a single NP (Fig. 3 and Supplementary Fig. S5). As shown in Fig. 3, in the two-NP case, the WNPs can penetrate through the membrane completely (Fig. 3a), whereas the RNPs cannot (Fig. 3d). These results are directly opposite to those found in the one-NP case. The ONPs and JNPs remain embedded in the membrane (Fig. 3b-c), the same as that in the one-NP case. We further increase NP quantity from two to four, and similar results as the two-NP case are observed (Fig. 3e-h). These observations clearly suggest that the increase of NP quantity may make the NP’s membrane penetration behavior totally different, and further confirm the complexity of the interactions between multiple NPs and the lipid membrane observed in the previous studies^13^,^46^. For simplicity, our following analysis will be concentrated on the two-NP case.

**Figure 4:**
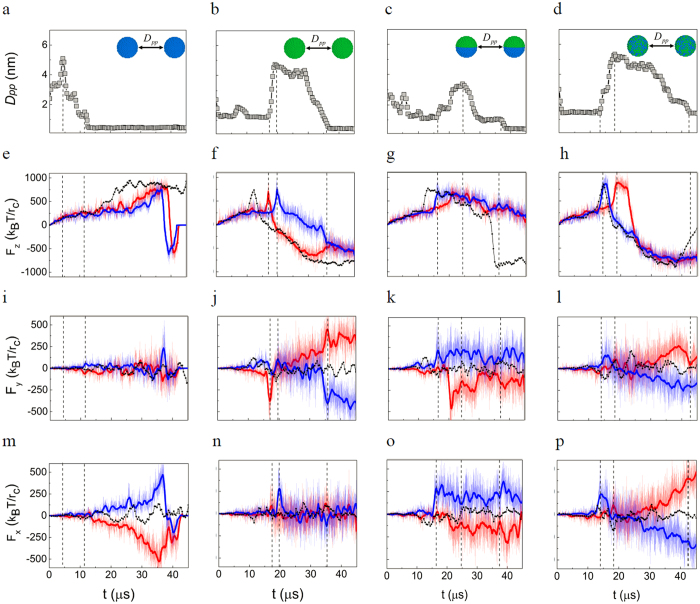
Effects of NP-membrane force on the cooperation of NPs in the penetration . (**a-d**), Typical changes of the distance between the two NPs in the particle penetration process into a membrane: **a**, WNPs, **b**, ONPs, **c**, JNPs, and **d**, RNPs. (**e-p**), The different components of NP-membrane forces in the penetration process: **e-h**, z components, **i-l**, y components, and **m-p**, x components. From left to right, figures correspond to the cases of WNPs, ONPs, JNPs, and RNPs, respectively. In order to clarify the changes, the corresponding central line of each force component profile is highlighted by the bold line with the same color: blue line stands for NP1 and red line represents NP2. For comparison, the corresponding force component profile in the one-NP case is also shown by using the black dotted line. The dashed lines emphasize some typical one-to-one correlations between the inter-NP distance changes and the NP-lipid membrane force components. The initial distance between the NPs is 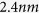. 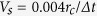.

Especially, it is worth stressing that the interesting cooperative ways of NPs in the penetration process are observed in our simulations, which can be manifested by the change in the inter-NP distance (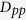): the WNPs always aggregate closely in the most penetration process, while other NPs follow an aggregation-dispersion-reaggregation mode (Figs. 3 and 4a-d). The packing state of NPs might affect the penetration. For example, aggregation of NPs possibly benefits the penetration. Compared with a single NP, the energy barrier of a two-NP cluster’s membrane insertion changes from 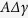 to 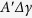, where 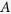 and 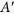 are the exposed surface area of a single NP and the two-NP cluster, respectively, and 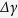 is the surface energy density difference between two typical NP-membrane interaction states: the NP stays on the membrane surface and it is inside the membrane. Since 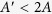, the doubled pulling forces of two-NP cluster may be high enough to overcome the energy barrier, which facilitates the NP cluster to pierce into/through the membrane. The penetration of WNPs shown in Fig. 3 may be an example of such a speculation. However, if we only follow this line, the translocation behaviors of ONPs, JNPs and especially RNPs would become rather “strange”. Note that, under the similar conditions, the insertion of these NPs into the membrane is easier than the WNP, and the RNP can even fully translocate through the whole membrane, namely the applied pulling force on a RNP is sufficient to surmount the energy barrier in a RNP’s membrane penetration. However, the complete penetration of ONPs, JNPs and even RNPs does not occur under the similarly doubled pulling forces, no matter what states these NPs stay (aggregation or dispersion, see Fig. 3). Additionally, the profile of resistance forces acting on the NPs provides a mechanical description of the translocation process of NPs. Compared with the one-NP case, it is found that the resistance force of each individual NP in the multiple-NP case becomes smaller in the most translocation process (Supplementary Fig. S6), although the NP quantity increases. All these results indicate again that the appearance of the second NP greatly changes the interaction mechanism of NPs with the membrane and consequently the penetration behavior of NPs.

The force between the NPs and the membrane (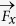, 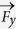, and 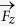) is undoubtedly crucial for the membrane penetration of NPs. By inspecting the “force spectrum” of NPs with the membrane, it is interestingly found that in the two-NP cases, the component(s) of NP-membrane force in the membrane plane direction (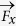 or 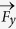 or both) obviously deviate(s) from zero, but in the one-NP case such components only fluctuate around zero (Fig. 4i-p and Supplementary Fig. S3e-h). In other words, the appearance of the second NP destroys the symmetry of the in-plane forces between the NPs and the membrane. Furthermore, the deviations of these force components have one-to-one correlations with the inter-NP distance changes of NPs (Fig. 4). We find that the aggregation of NPs always occurs when the in-plane force components of two NPs (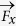 or 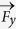 or both) begin to simultaneously deviate. But if only one NP’s force component deviates obviously while the other’s does not, the aggregation of NPs is disrupted and rapid dispersion occurs. These findings suggest that it is the breaking of symmetry of in-plane NP-membrane force that induces the cooperative behavior of the NPs in the penetration.

**Figure 5:**
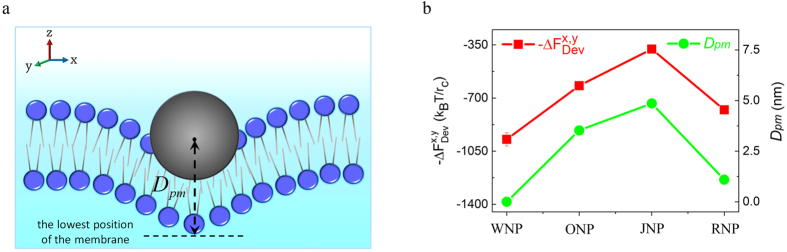
Effect of changes of in-plane NP-membrane force components on out-of-plane penetration . (**a**), Sketch of the membrane penetration degree of the NP, 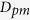. (**b**), Changes of 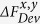 and 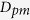 of different types of NPs at the final stage (44.5~45.0 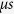) of the translocation (ONPs, JNPs and RNPs) or at the fully penetration moment (WNPs) in the simulations. The initial distance between the NPs is 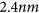. 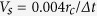.

Importantly, we find that the penetration progress of NPs in the membrane is also tightly associated with the deviations of in-plane force components between the NPs and the membrane. Here we define the deviation degree of force as 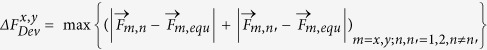 where 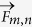 is the “m” component of the force of the “nth” NP and 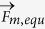 is the mean value of the “m” component of the force in the corresponding one-NP case (~zero in all cases). Also, the membrane penetration degree of the NP is taken as 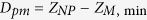 where 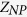 is the z-position of the NP center-of-mass and 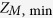 is the lowest z-position of the membrane in the interaction region (Fig. 5a), and thus the smaller 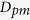 indicates that the NP is more likely to realize a complete penetration and when 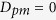, the NP exactly finishes the whole membrane translocation process. As shown in Fig. 5b, it is really found that the deviation degree of in-plane force strongly affects the out-of-plane translocation behavior of NPs: the larger 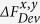 is, the smaller 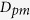 is. Therefore, in the multiple-NP case, the changes of NP-membrane interaction within the membrane plane help the translocation of NPs in the direction perpendicular to the membrane plane.

On the other hand, there exists a close link among three components of NP-membrane force in the NPs’ membrane translocation process. That is, the membrane is deformed under the effect of 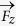, and in turn, the deformation causes the deviation of 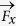 or 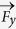 and the cooperation of NPs. Take the two-RNP case as an example, the membrane is deformed with the increase of 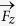 (Figs. 3i,4h). However, the deformation profile of the membrane around each individual RNP (in the two-NP case) loses symmetry compared with that in the one-NP case (Fig. 3i). It is such a change in membrane deformation that induces the deviations of in-plane force components of RNPs. If the membrane deformation near each RNP is similar, such as the initial stage of RNP’ translocation (Fig. 3i), 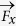 (and 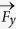) of both RNPs deviates roughly symmetrically and simultaneously (Fig. 4l-p), and thus aggregation of the RNPs appears. However, the similarity could be disrupted with the change of 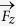, such as the situation that one RNP pierces into the membrane (Fig. 3j). On this occasion, the dissimilarity in membrane deformation makes the deviations of 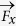 (and 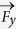) of two RNPs different (Fig. 4l-p), and consequently the dispersion of RNPs. Moreover, the deviation degree of in-plane force components of RNPs is influenced by the membrane deformation degree or the magnitude of 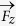. After both RNPs are totally included in the membrane, the membrane deformation profiles near each RNPs become similar again (Fig. 3k). Thus the deviations of 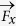 (and 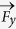) of the two RNPs become similar as well. At this stage, the membrane is deformed dramatically because of the increase of 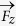 in magnitude compared with the initial stage. Accordingly, the deviations of 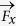 (and 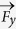) are also notable and finally facilitate the membrane translocation of the RNPs (Fig. 5).

The surface chemistry of the NP also affects such a link. Under the similar 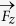, the membrane deformation induced by different types of NPs is different. For the WNPs, both particles tend to stay on the membrane surface due to the hydrophilic surfaces (Fig. 3). Thus the deviations of 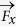 of two WNPs are always increasing in a similar way (Fig. 4m). But for other NPs, the ease of insertion into the membrane disrupts the increasing of deviations of 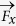 or 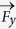 (e.g., Fig. 4p). As a result, both the cooperative way of NPs and the progress of membrane penetration are changed (Figs. 4,5). Overall, it is the joint effect of 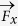, 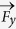 and 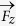, not just 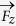 as in the one-NP case, that dominates the NPs’ penetration behavior.

Similar to the one-NP case, the applied 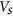 has a strong impact on the penetration of multiple NPs and their cooperation as well. It is found that, under a reduced applied velocity 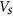 (e.g., 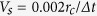), the membrane deformation becomes gentle, and accordingly only aggregation or aggregation-dispersion of NPs is observed (Supplementary Figs. S7-8). Furthermore, if the individual NPs are driven by different velocities during the penetration, their translocation becomes more complicated (Supplementary Figs. S9). In this situation, the NPs always translocate into/through the membrane in a certain order due to the different 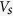, and thus the NPs are not always in the similar positions relative to the membrane at the end of simulations. However, the similar cooperative ways of NPs (aggregation or aggregation-dispersion-reaggregation) are still observed in the penetration process (Supplementary Figs. S9). Based on our simulations, the velocity 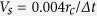 is a threshold, which is within a typical experimental range for the delivery of NPs as nanocarriers under the external fields^47^,^48^. Only those NPs whose applied velocities are larger than this threshold value might completely translocate through the membrane, and the successful penetration probability of the NPs increases with the increase of 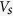
(Supplementary Fig. S10)^20^,^49^. Even under a larger 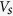, however, the NPs still adopt the similar cooperative modes in the penetration process (Supplementary Fig. S10). All these results confirm the ubiquitous of the cooperative transmembrane penetration behavior of multiple NPs. Besides, the membrane tension, which has been demonstrated by both simulations and theoretical studies to be crucial for the interaction mode of NPs with the membrane^41^,^50^, also possibly affects the cooperative penetration of NPs by changing the packing state of lipids in the bilayer or the tendency of membrane deformation. However, we interestingly find that the change of the initial distance between the two NPs (from 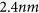 to 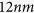) in the simulations has only a small influence on the translocation dynamics of the NPs (Supplementary Fig. S11).

In addition, based on the previous studies^41^,^51^,^52^, the spontaneous change of inter-NP distance of NPs is directly related with the “effective” interaction induced by the membrane deformation. Especially, the characters of this interaction^53^, such as the switch of its sign^54^, and its dependence on the subtle deformation profile of the membrane and lipid packing state^54^,^55^, are still considered as an open question to date. Here, our results could give a new insight into this point. In our simulations, the aggregation-dispersion-reaggregation of NPs is observed, in which the aggregation/reaggregation reflect that the membrane-induced interaction is attractive, but the dispersion of NPs suggests a transition of this “effective” interaction from attractive to repulsive. As mentioned above, the packing state of NPs (i.e., aggregation or dispersion) in the membrane is not merely a result of membrane deformation. The situations of the membrane deformation near the NPs (similar or dissimilar) and the symmetry of the NP-membrane force fundamentally determine the character of such an “effective” membrane-induced interaction. For example, if the membrane deformation profiles near the two NPs are similar and 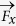 or 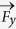 of both NPs deviates synchronously, the interaction is attractive; but when the deformation profiles near the two NPs are different, it is repulsive. Therefore, the nature of the “effective” membrane deformation-induced interaction between NPs is the breaking of symmetry of the NP-membrane force between the NPs and the membrane.

## Conclusions

In summary, the cooperative penetration behavior of multiple NPs into/through a lipid membrane is investigated by computer simulations. We find that the particle quantity plays key roles in the nanoparticle penetration process. However, the increase of particle quantity does not always provide help for the NPs to penetrate a membrane due to the huge change in the NP-membrane interaction mechanism. Therefore, in experiments or biomedical applications, the concentration of the NPs should be carefully chosen according to their surface chemistry. Additionally, the coupling between the particle quantity and surface chemistry of the NPs further complicates the penetration process. Some interesting collective behaviors among NPs, such as aggregation, aggregation-dispersion, or even aggregation-dispersion-reaggregation, are observed during the particle penetration process. These behaviors, which are caused by the changes of the NP-lipid membrane forces in the membrane plane direction, strongly affect the penetration behaviors of the NPs. Our results are very helpful for the applications of NPs in drug or gene delivery system, and understanding of the interaction mechanism between NPs and cell membrane.

## Methods

Dissipative particle dynamics (DPD) is a coarse-grained modeling technique. In the simulations, the dynamics of the coarse-grains (beads) obeys Newton’s equation of motion. The inter-bead interactions include conservative force (
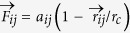
), dissipative force and random force. Here 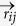 is the distance between beads *i* and *j*, *r*_*c*_ is the cut-off radius of the force, and *a*_*ij*_ is the maximum possible repulsive interaction between the two beads. The simulations are performed in the NVT ensembles at the temperature 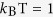 and time step 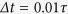 (where 
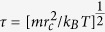
 is the characteristic time scale of the model), with periodic bound conditions imposed in the three directions.

Our simulation model consists of a tensionless lipid membrane immersed in sufficient water. The simulation box size (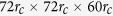) is chosen to be sufficient large to ensure computational convergence. Each lipid molecule is coarse-grained as a linear chain with two hydrophilic head beads and five hydrophobic tail beads. Both the pair-wise bonding energy and three-body bond angle bending energy are described by harmonic spring potentials with the spring constants 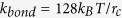 and 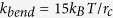, respectively. In addition, four types of NPs are included in the simulation box (Fig. 1). According to the character of beads, we set 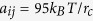 between the hydrophilic and hydrophobic beads, and 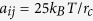 between the beads with the same character. By mapping the characters of DMPC molecules in the bilayer (diffusion, area per lipid and bilayer thickness) with our lipid model, we can obtain the reduced DPD length and time units are 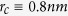
and 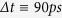, respectively^56^.

Prior to the simulations, the NPs are docked above the bilayer (the initial distance between them is 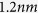). The system is then equilibrated while keeping the locations of the NPs fixed. Additionally, as done in previous simulations^9^,^16^,^26^,^33^,^35^, a moving spring force with velocity 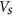 along the z-direction is applied on the center-of-mass of the NP to guide its penetration, which is similar to the membrane translocation process of the NP aided by electric force, AFM tip or by injection method in experiments^6^,^15^,^17^,^18^. A larger 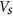 corresponds to a larger driving force for the NP penetration. All simulations are carried out with at least 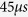
(5 × 10^5^ simulation steps) and three independent runs to ensure the computational convergence and consistency.

## Additional Information

**How to cite this article**: Zhang, H. *et al*. Cooperative Transmembrane Penetration of Nanoparticles. *Sci. Rep.* **5**, 10525; doi: 10.1038/srep10525 (2015).

## Supplementary Material

Supplementary Information
