# Supplementary material for: Cooperative Transmembrane Penetration of Nanoparticles
Source: Sci Rep. 2015 May 27;5:10525. doi: 10.1038/srep10525 (PMC4444962; doi:10.1038/srep10525)
Supplement: Supplementary Information [file srep10525-s1.doc]

**Supplementary Information**

**Cooperative Transmembrane Penetration of Nanoparticles**

Haizhen Zhang^1^, Qiuju Ji^1^, Changjin Huang^2^, Sulin Zhang^2^,

Bing Yuan^1^, Kai Yang^1^ and Yu-qiang Ma^1, 3^

^1^ Center for Soft Condensed Matter Physics and Interdisciplinary Research, Soochow University, Suzhou, 215006, China.

^2^ Department of Engineering Science and Mechanics, The Pennsylvania State University, University Park, Pennsylvania 16802, United States.

^3^ Collaborative Innovation Center of Advanced Microstructures and Department of Physics, Nanjing University, Nanjing, 210093, China.

Correspondence and requests for materials should be addressed to K.Y. (email: yangkai@suda.edu.cn) or Y.Q.M. (email: myqiang@nju.edu.cn).

**Supplementary Information 1**：More trajectories of the translocation behaviors of a single NP (Supplementary Fig. S1), the typical mechanical description of the penetration (Supplementary Figs. S2 and S3), and the translocation behaviors of a single NP under a changed driving force (Supplementary Fig. S4).

a

b


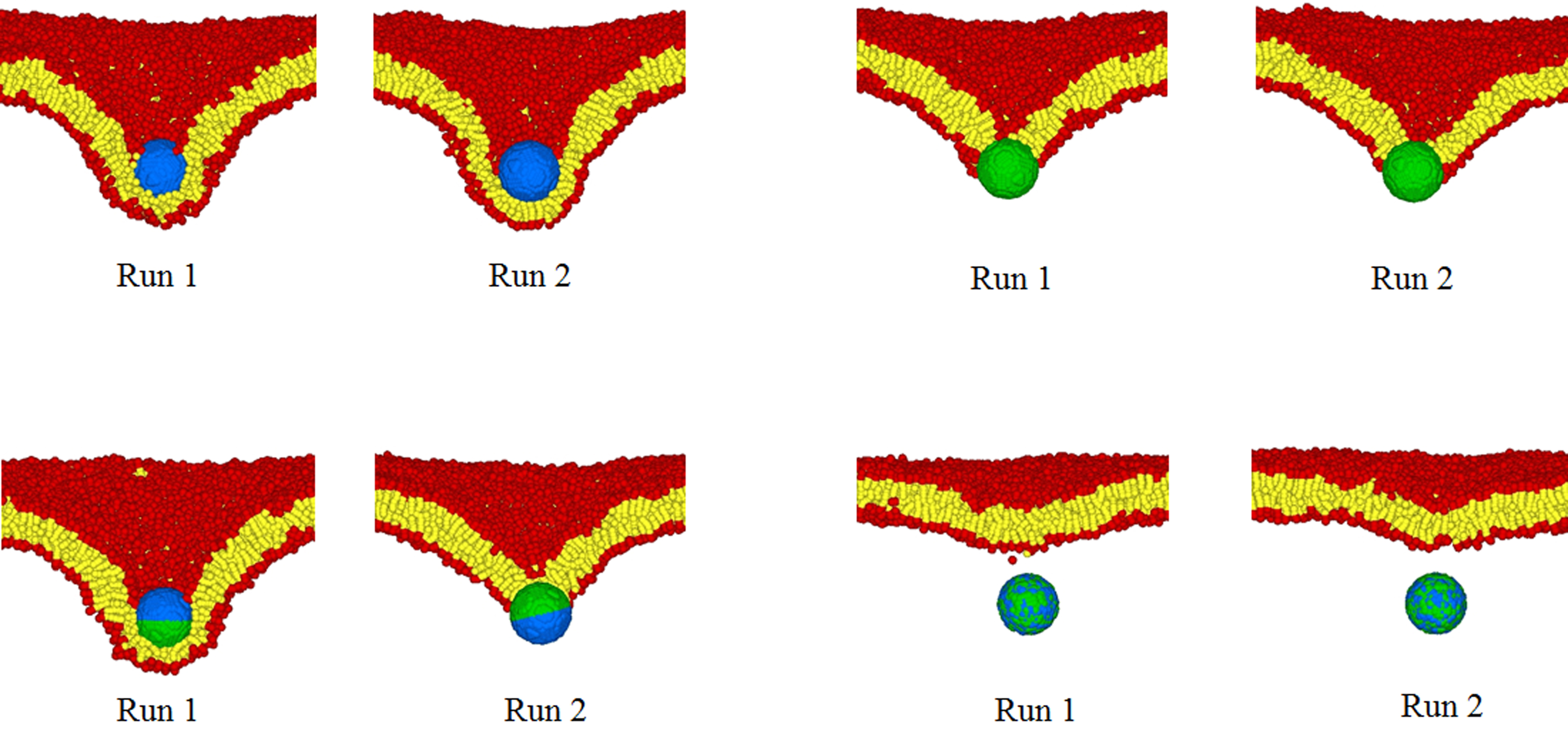


c

d

**Supplementary Fig. S1** More trajectories (final snapshots) of the translocation behaviors of one single NP. (**a**), WNP. (**b**), ONP. (**c**), JNP. (**d**), RNP. The word of “Run 1 (2)” in the figure means the order of independent run. $t=45\mu s$, $V_{s}=0.004r_{c}/\Delta t$.

More trajectories of the membrane translocation of one single NP are shown in Supplementary Fig. S1. These independent simulations confirm the results shown in Fig.2 in the text: at the end of the simulations, the WNP stays on the membrane surface, the JNP and ONP are inside the membrane, while the RNP totally penetrates through the membrane. Furthermore, in the one independent run of JNP’ translocation (Supplementary Fig. S1c, Run 1), a different interaction configuration of JNP and the membrane is observed, which is the result of the complicated rotation motion of JNP in the penetration process[^1^](#_ENREF_1). Actually, such a configuration is also observed in the other independent runs of JNP’ translocation (Supplementary Fig. S1c, Run 2 and Fig. 2c in the text): after this interaction state, the JNP rotates itself rapidly under the moving spring force, to realize the further translocation inside the membrane. As a result, it reaches the bottom monolayer of the bilayer membrane from the top surface of the membrane; meanwhile, the deformation of the membrane becomes gentle (as shown in Supplementary Fig. S1c, Run 2).


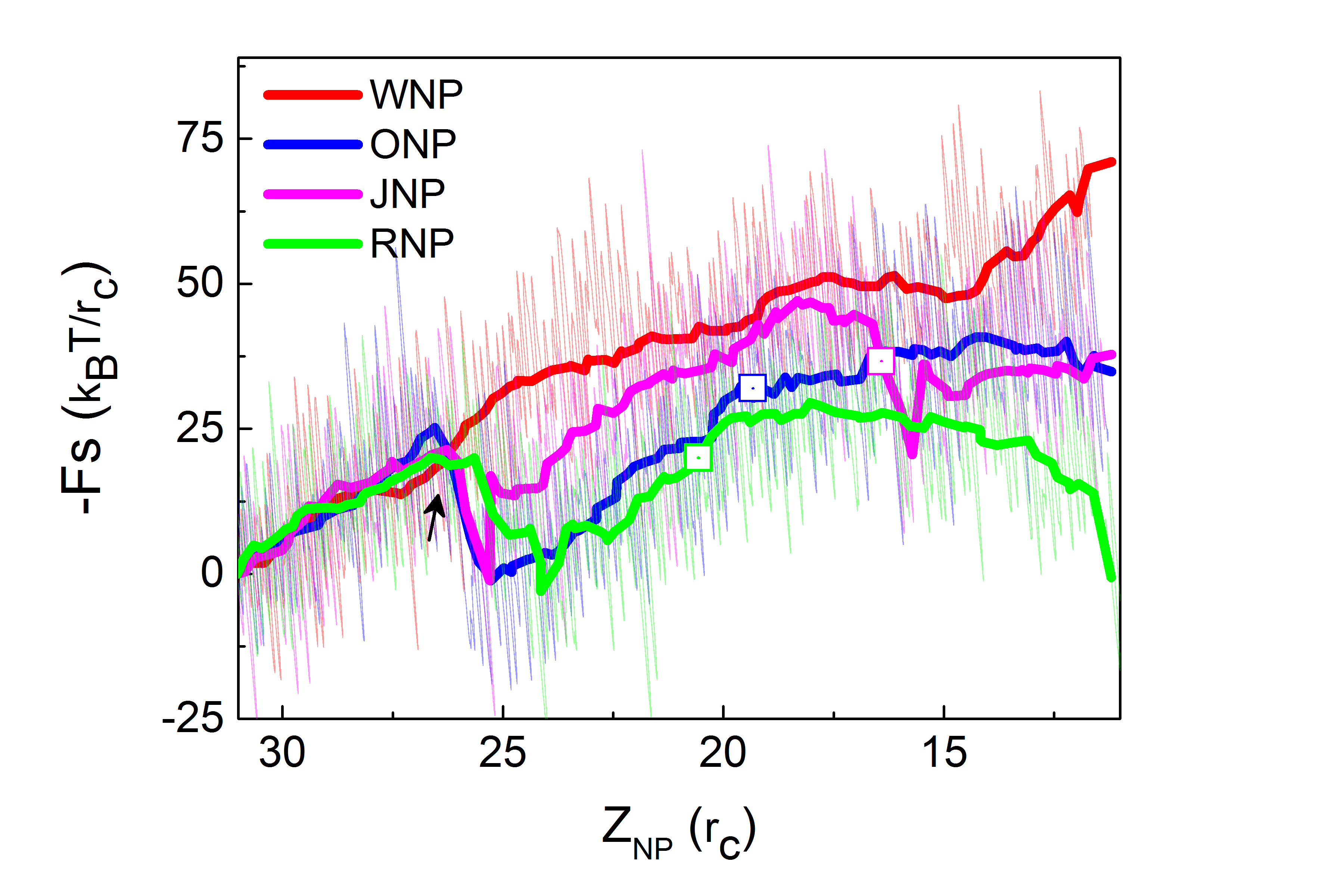


**Supplementary Fig. S2** Representative resistance force acting on NP during the translocation process. In order to clearly clarify the changes of force, the corresponding central line of each force profile is highlighted by the bold line with the same color. Z_NP_ is the position of the center-of-mass of the NP (the zero of Z axis is at the bottom of the box), and at the beginning of the simulation the NP is placed on the top of the membrane plane. Arrow indicates the location where the NP (ONP, JNP and RNP) starts to translocate into the membrane. Hollow squares are used to mark the position of the center of the membrane. $V_{s}=0.004r_{c}/\Delta t$.

The NP’s surface chemical property plays a key role in the NP’s translocation process, which can be reflected by the changes of the resistance force acting on the NP. Because the membrane penetration of NP is guided by a moving spring, whose velocity, $V_{s}$, is constant and rather small (that is, the NP’s move in every simulation step is also very small), we could roughly consider that the resistance force acting on the NP during its penetration is equal in the magnitude but opposite in the direction to the driving force of particle penetration ($\vec{F}_{s}$). As shown in Supplementary Fig. S2, the resistance forces are different for various types of NPs: the resistance force of the WNP is the largest, and that of the RNP is the smallest. Furthermore, for the WNP, its resistance force always increases with the proceeding of WNP’s translocation. But for the other three types of NPs, the resistance force has a sharp drop to zero or even a negative value after the NP’s initial contact to the lipid membrane (Supplementary Fig. S2, indicated by the arrow), indicating that the NP can easily or even spontaneously insert into the membrane. However, after the NP’s insertion into the membrane, the resistance force still increases with the further translocation of the NP until the NP is near the membrane center. Then the resistance force of the ONP or JNP roughly keeps in the maximum value, but for the RNP, its resistance force finally reduces to near zero after a period of time when the RNP reaches the maximum (due to inertia, the RNP’s resistance force can even be a small negative value at the end of the simulation). This drop, based on the detailed translocation process of RNP, indicates the transition of complete penetration of RNP from the inside of the membrane to the outside of the membrane.

a b c d


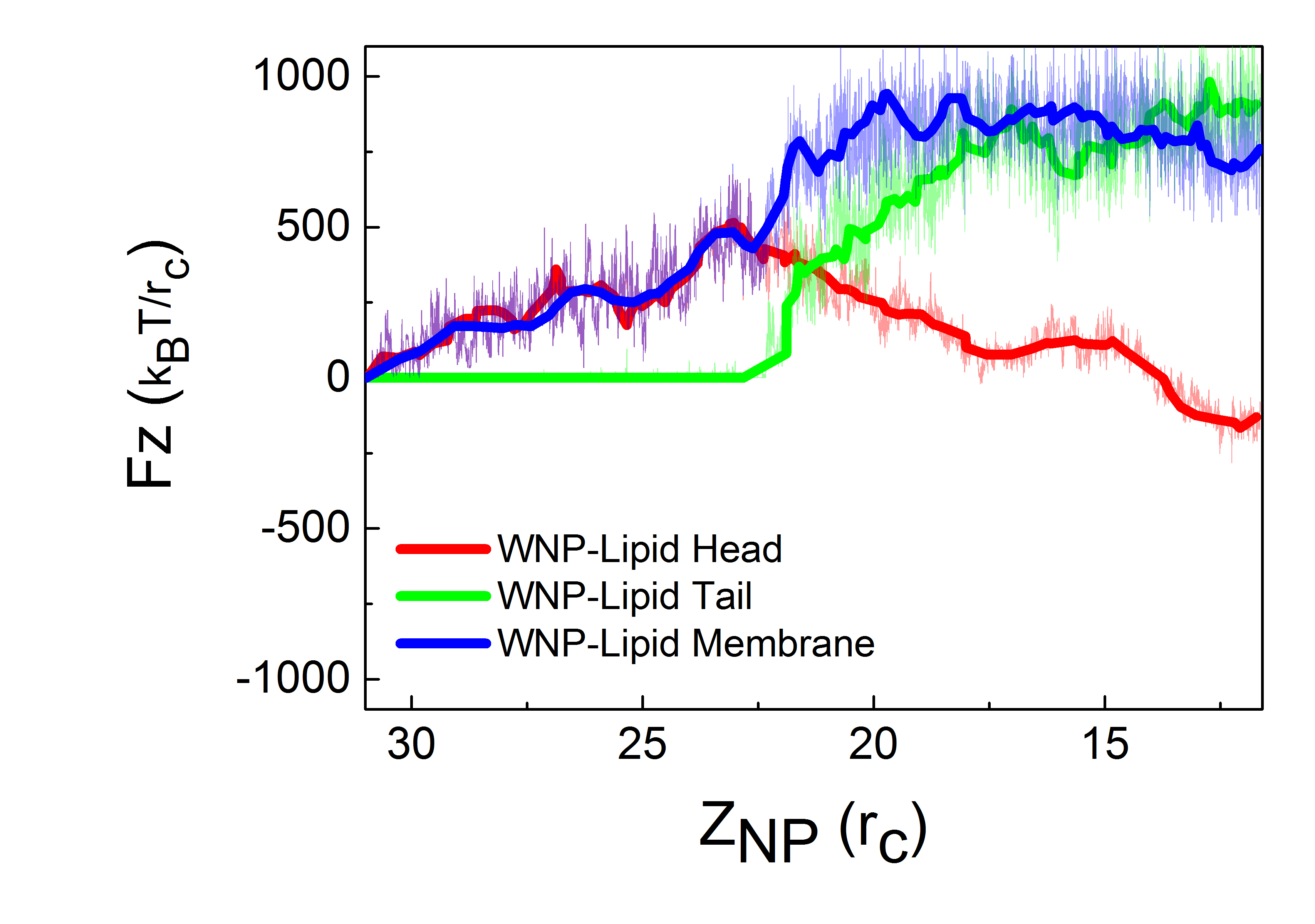

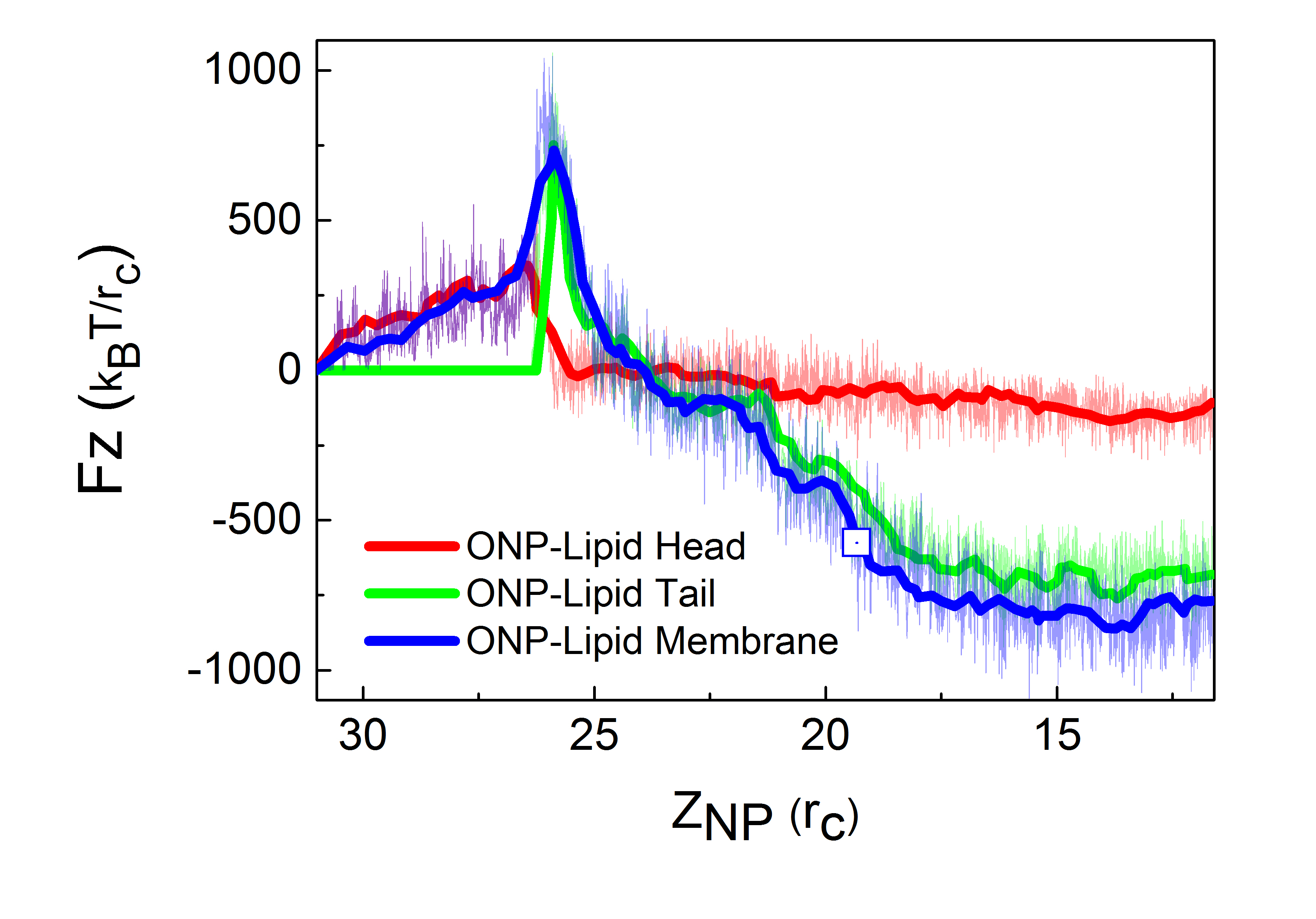

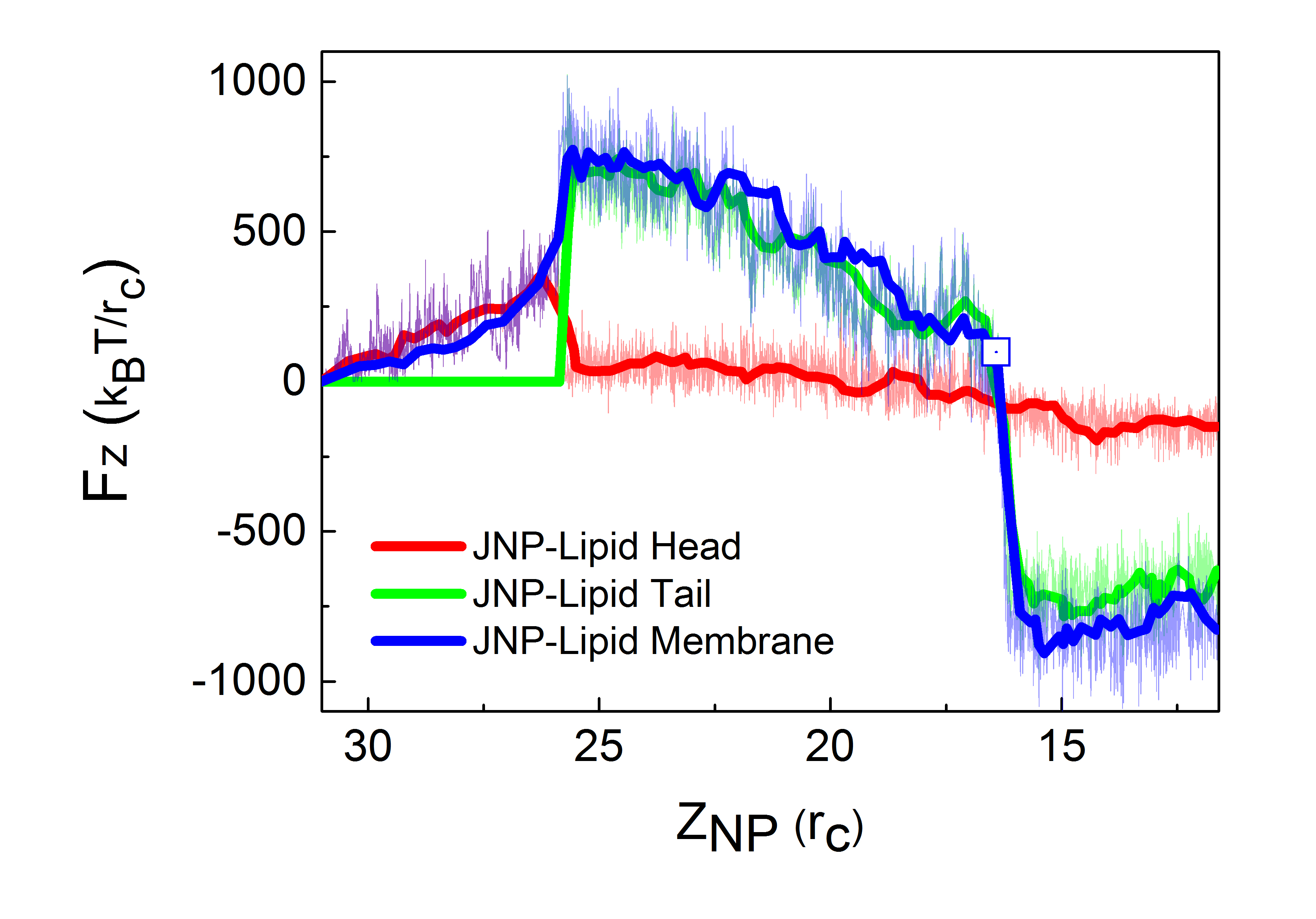

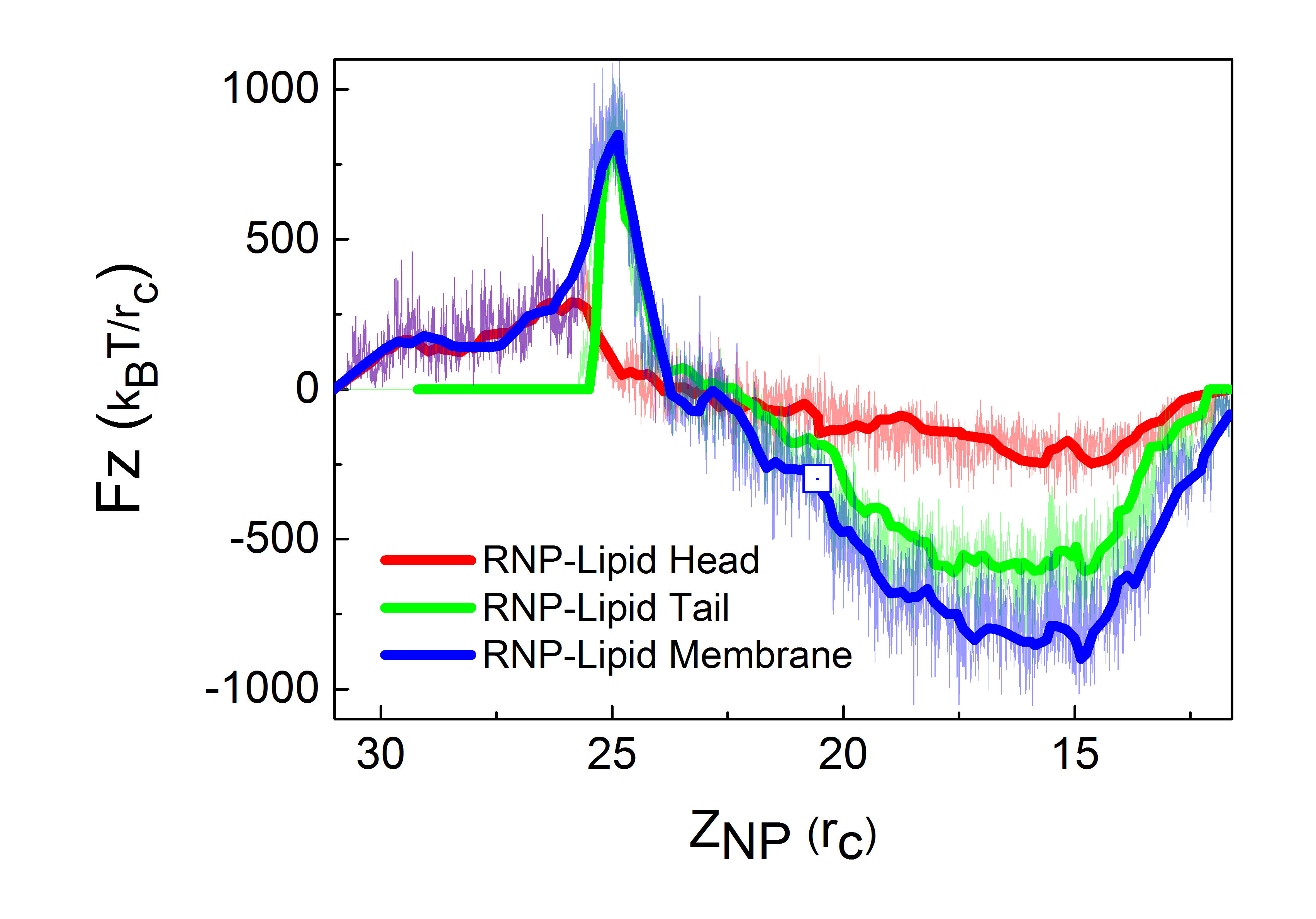


e f g h


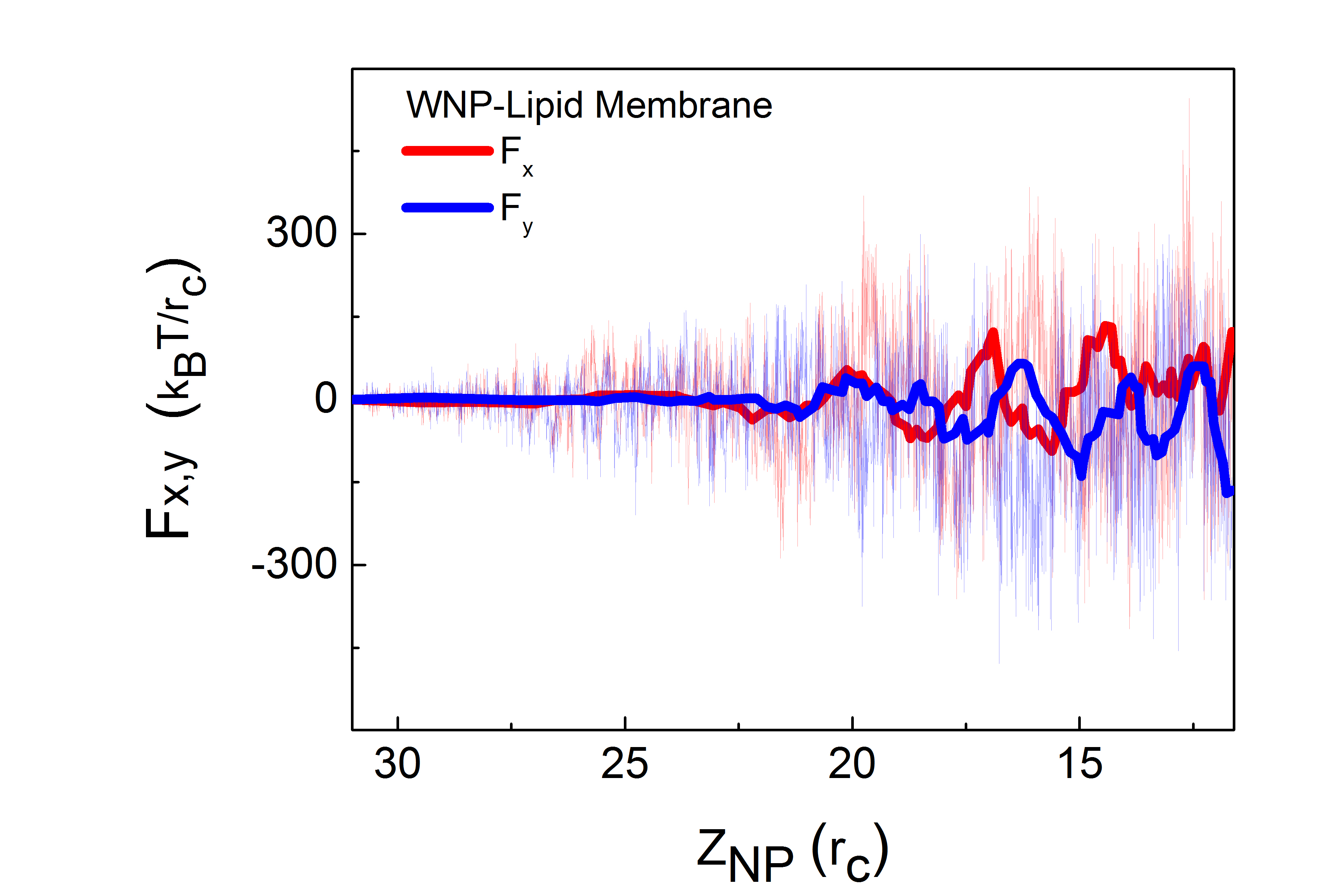

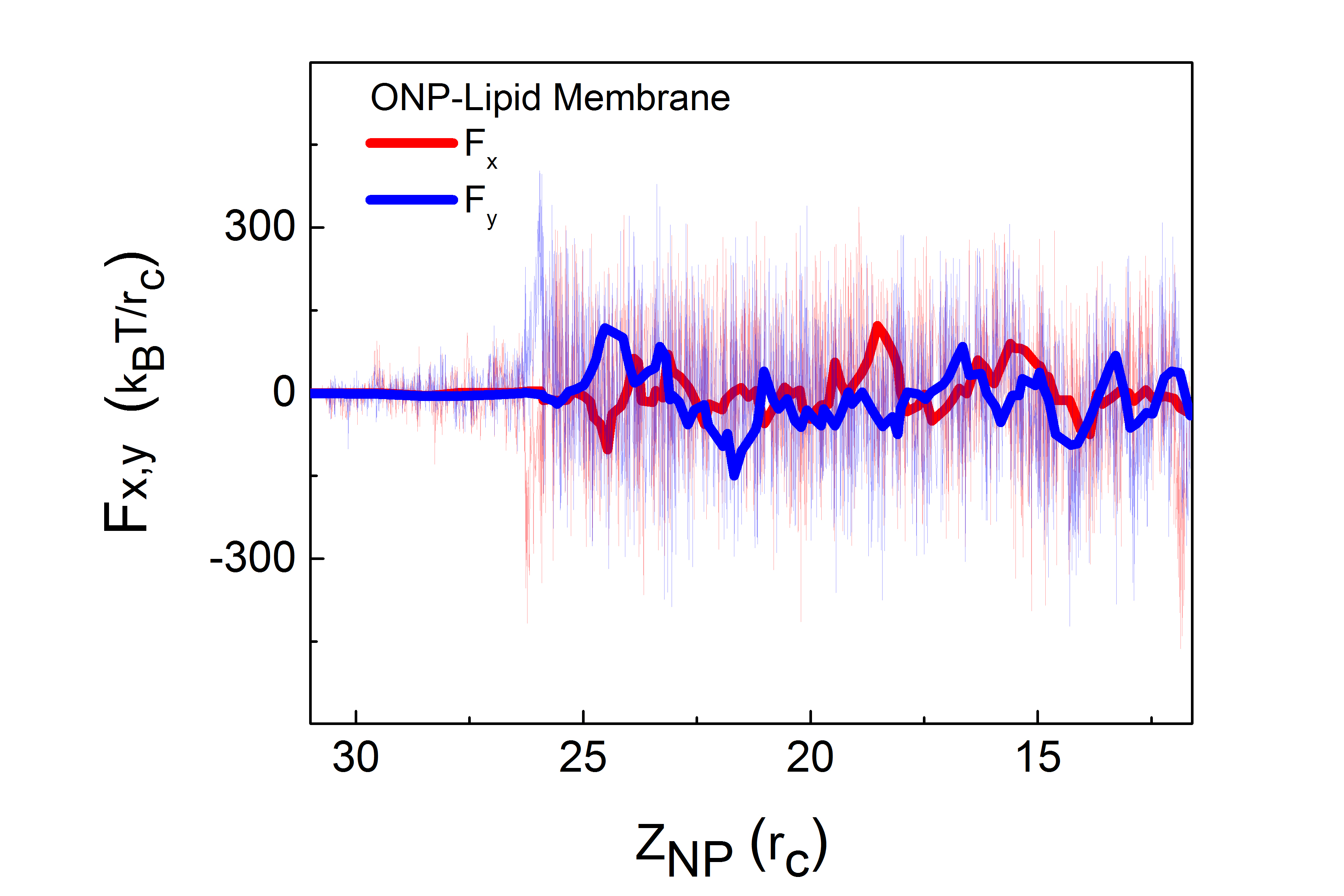

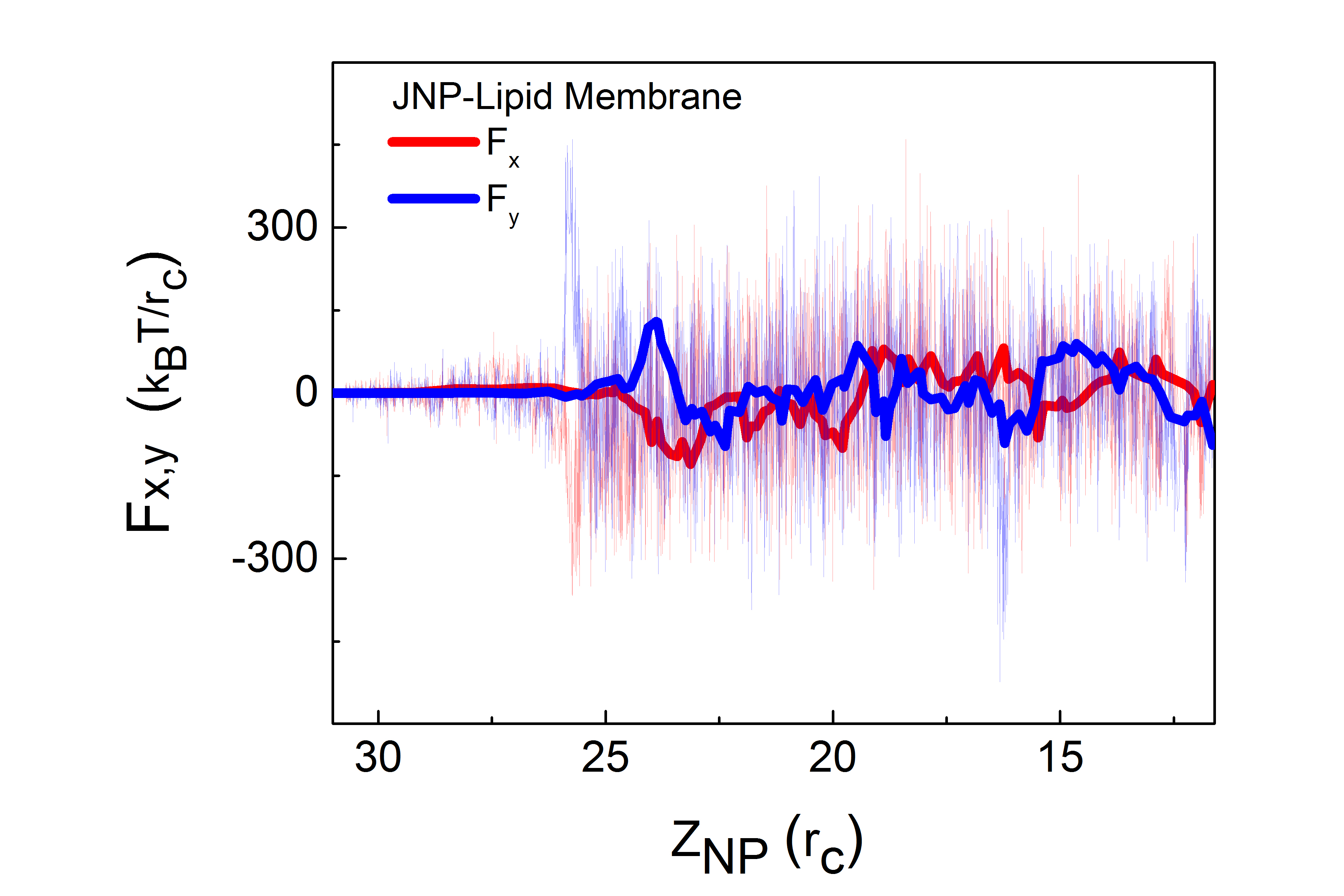

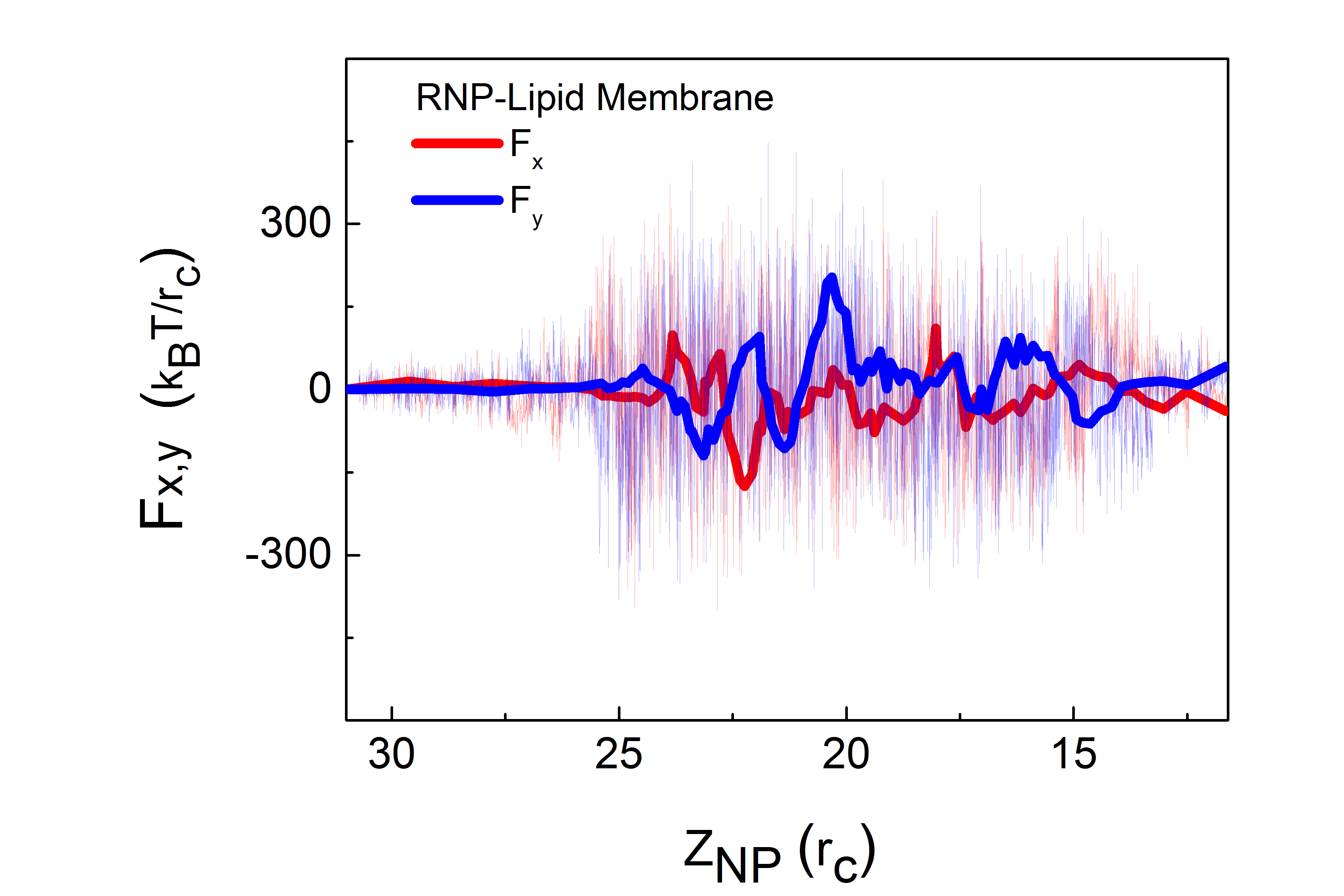


**Supplementary Fig. S3** Evolution of forces between the lipid membrane and the NP with different surface chemical properties (light color lines), respectively. (**a-d**), $\vec{F}_{z}$; (**e-h**), $\vec{F}_{x}$ and $\vec{F}_{y}$. In order to clearly clarify the changes of force, the corresponding central line of each force profile is highlighted by the bold line with the same color. Hollow squares are used to mark the position of the center of the membrane. $V_{s}=0.004r_{c}/\Delta t$.

The changes of NP-lipid membrane force profiles, especially their z components, reflect the influence of surface chemical properties of a NP on the translocation into/through a lipid membrane. First, the difference between NP-lipid head force profile and NP-lipid tail force profile indicates that the NP interacts with the hydrophilic lipid heads and hydrophobic lipid tails of the membrane in a consecutive manner during the translocation process (Supplementary Fig. S3a-d): at the initial stage, the NP only interacts with the lipid heads; however, once the NP starts to contact lipid tails, the force between the NP and lipid heads reduces to near zero. In addition, the interaction between the NP and lipid tails is strongly influenced by the surface chemical properties of the NP. Due to the hydrophilic surface of the WNP, the NP-lipid tail force increases gradually with the translocation of the WNP (Supplementary Fig. S3a) and even its interaction with the surrounding water molecules. As a result, the WNP tends to stay on the membrane surface. But for the ONP, JNP and RNP, a sharp increase in the force profile appears when they pass through the lipid head layer of the membrane (Supplementary Fig. S3b-d). Note that, in the DPD technique, all interactions between beads are repulsive. Thus the sharp increases in the NP-lipid tail force profiles of the ONP, JNP, and RNP indicate the easy entering of these NPs into the membrane. After the NP’s insertion into the membrane, the direction of force reverses, which reflects the tendency of these NPs to stay inside the membrane. According to the free energy calculation presented by Li and Gao, these changes are caused by the hydrophobic attraction between the NP surfaces and the lipid tails. Therefore, the ONP and JNP are trapped inside the membrane bilayer (Supplementary Fig. S3b-c). Moreover, the random surface chemical pattern of RNP could undermine the hydrophobic attraction to some extent (Supplementary Fig. S3d), which makes the RNP relatively easily pass through the membrane.

In addition, in the translocation process of all types of NPs, the x and y components of the NP-membrane force ($\vec{F}_{x}$ and $\vec{F}_{y}$) only fluctuate around the zero, as shown in Supplementary Fig. S3e-h. Therefore, it is suggested that, for the one-NP case, the penetration of NP is mainly determined by $\vec{F}_{z}$, not $\vec{F}_{x}$ and $\vec{F}_{y}$.


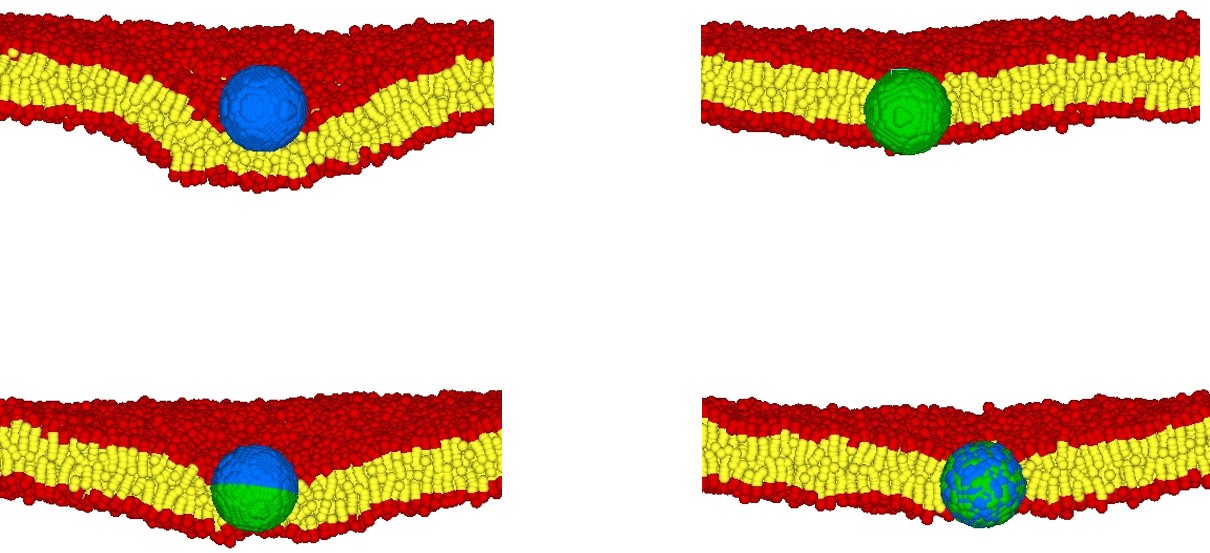


a

b

c

d

**Supplementary Fig. S4** Different translocation behaviors of the four types of NPs. (**a**), The one-WNP case. (**b**), The one-ONP case. (**c**), The one-JNP case. (**d**), The one-RNP case. $t=45\mu s$, $V_{s}=0.002r_{c}/\Delta t$.

The applied velocity $V_{s}$ is important for the NP penetration process. This velocity determines whether the pulling force is large enough to overcome the energy barrier for the NP in the translocation process (e.g., the one in the WNP’s insertion into the membrane and the one in the escape process of ONPs from the membrane interior). We find that when $V_{s}=0.002r_{c}/\Delta t$, none of the NPs can pass through the membrane: the WNP remains on the outer surface of the membrane, while the other three NPs are all trapped in the membrane (Supplementary Fig. S4).

**Supplementary Information 2**：More trajectories of the translocation behaviors of multiple NPs (Supplementary Fig. S5), typical changes of resistance forces of NPs during the membrane penetration (Supplementary Fig. S6), and the translocation behaviors of multiple NPs under a changed driving force (Supplementary Figs. S7-10) or various initial inter-NP distances (Supplementary Fig. S11).

a

b


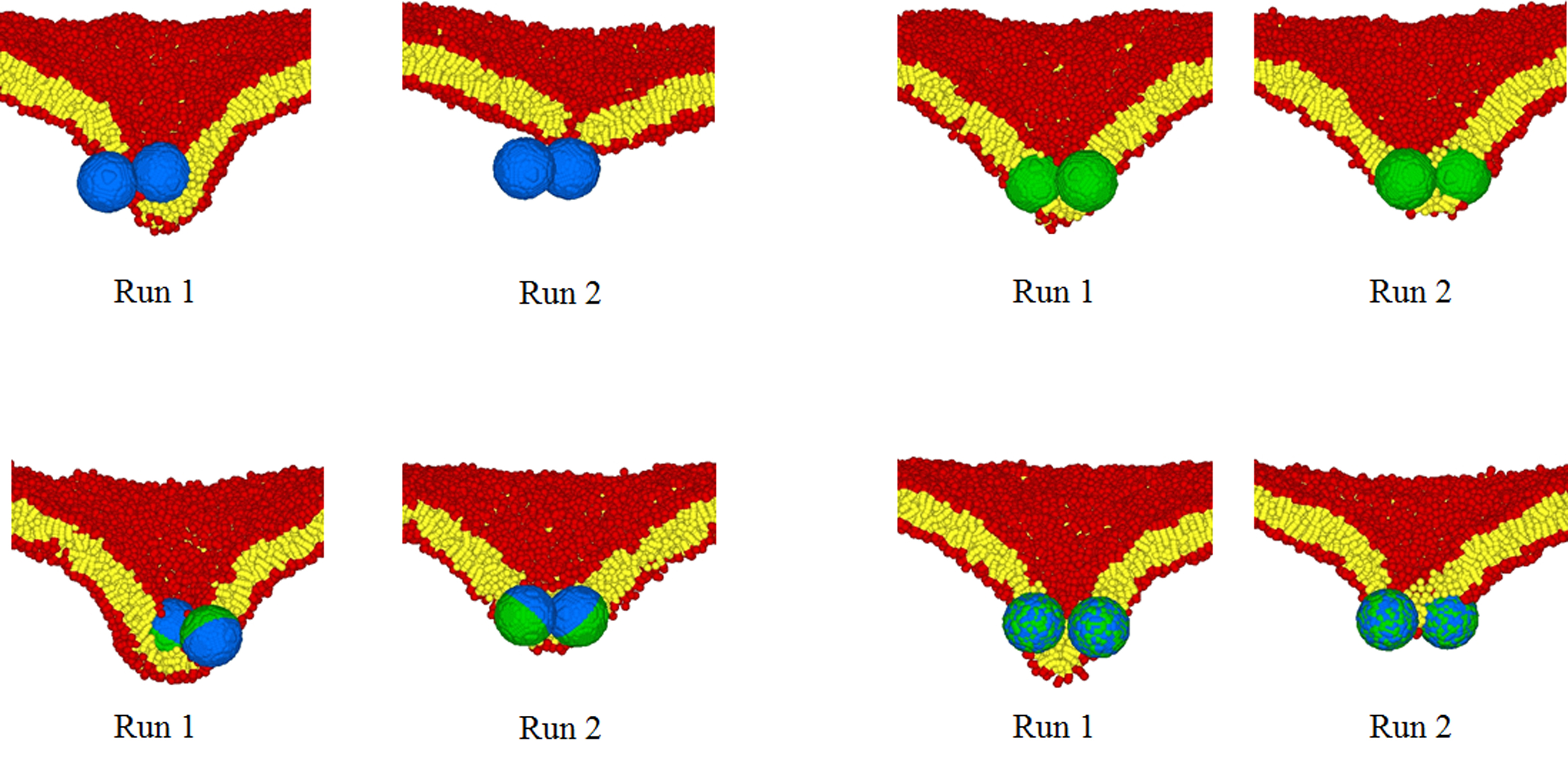


c

d

**Supplementary Fig. S5** More trajectories (final snapshots) of the translocation behavior of two-NP case. (**a**), WNPs; (**b**), ONPs, (**c**), JNPs, (**d**), RNPs. The word of “Run 1 (2)” in the figure means the order of independent run. $t=45\mu s$, $V_{s}=0.004r_{c}/\Delta t$.

In Supplementary Fig. S5, more trajectories of the translocation behaviors of two NPs are shown. The results shown here are similar as those in Fig. 3 in the text: for the WNPs, at least one WNP can completely penetrate through the membrane at the end of the simulations; for the ONPs and JNPs, they are trapped in the membrane; for the RNPs, no successful penetration behavior is observed. The different configuration of JNPs with the membrane shown in Supplementary Fig. S5c (Run 1) is still caused by the rotation of JNP, as mentioned in Supplementary Fig. S1.


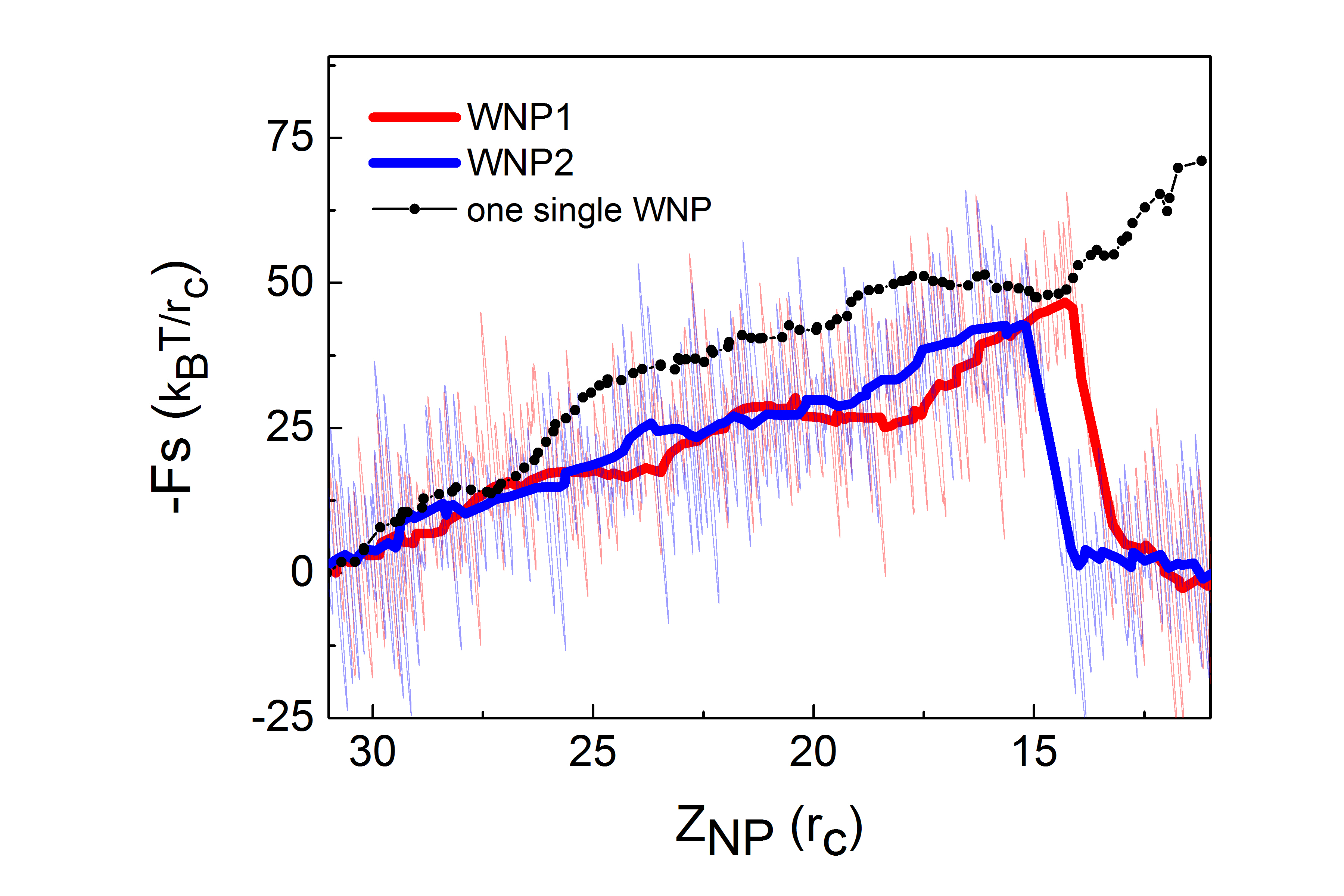

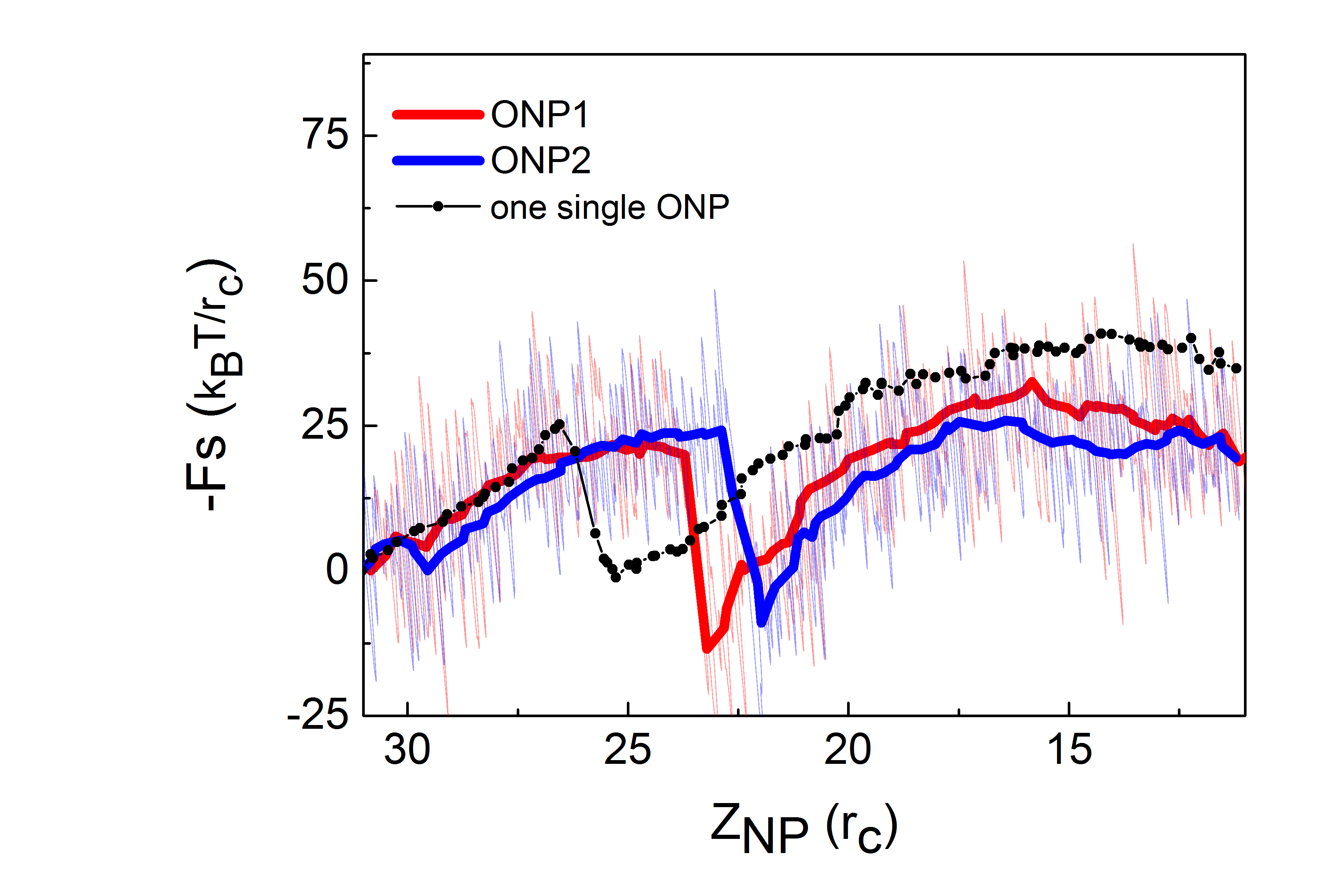


a

b

c

d


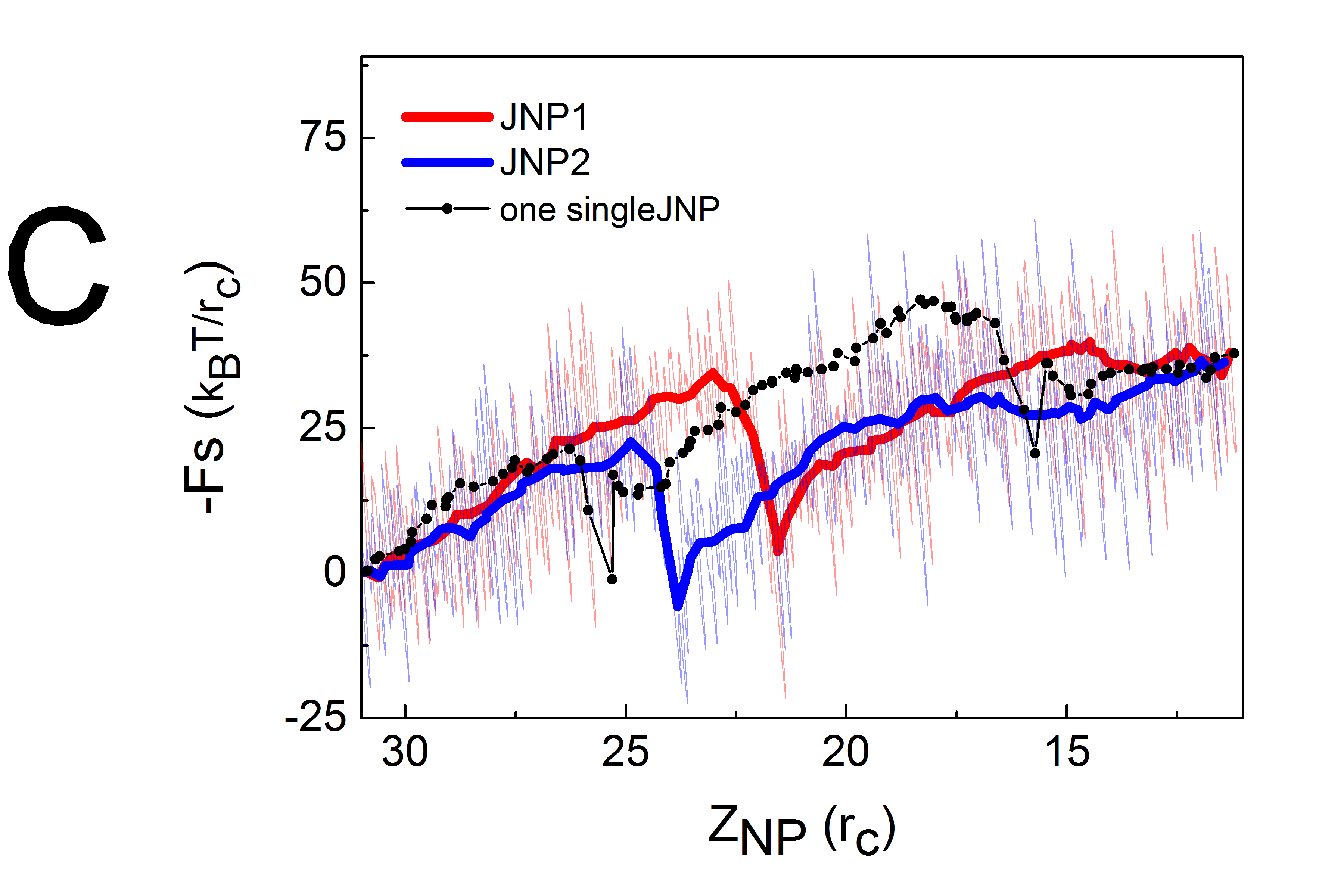

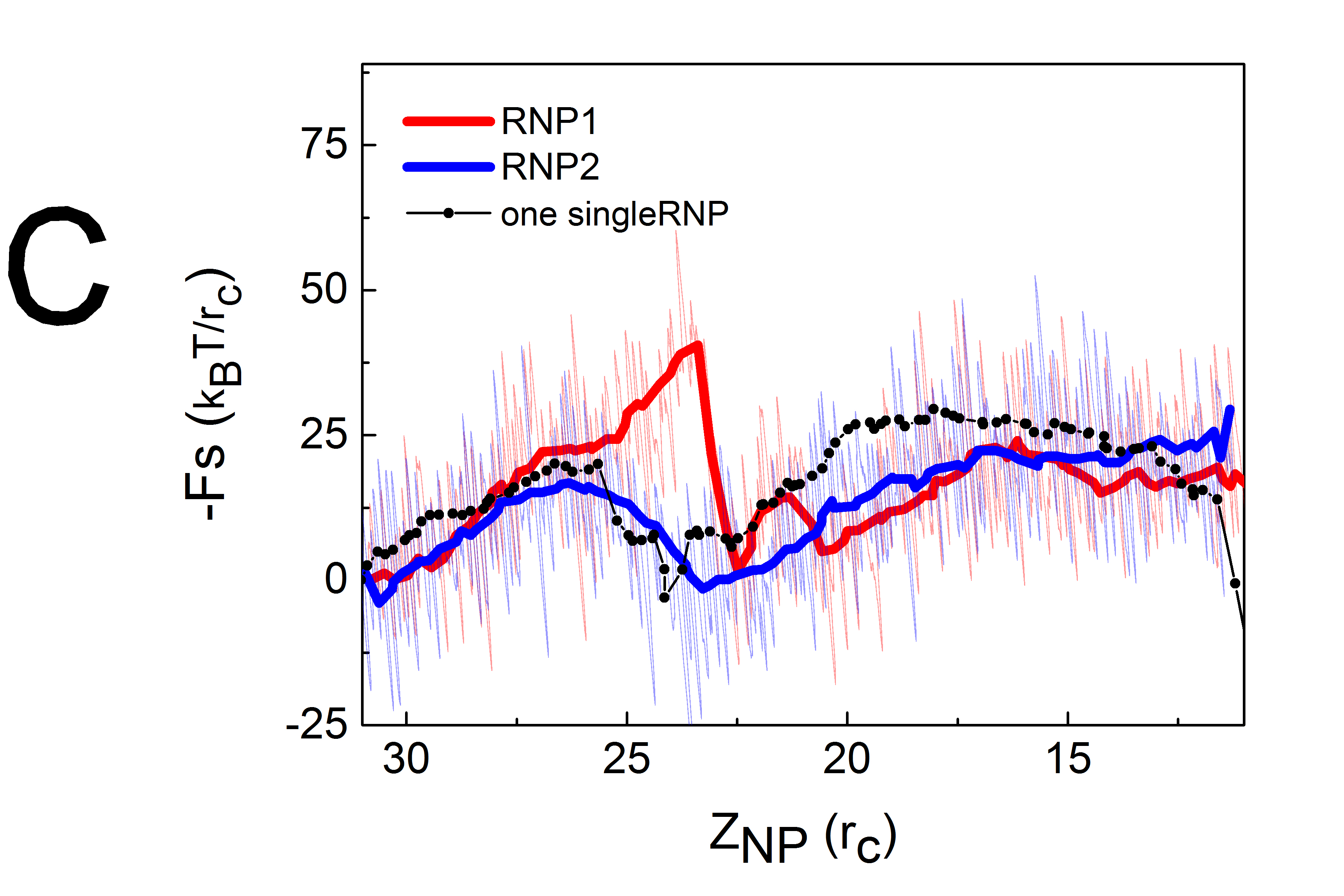


**Supplementary Fig. S6** Representative resistance forces acting on NPs during their translocation process. (**a**), The two-WNP case. (**b**), The two-ONP case. (**c**), The two-JNP case. (**d**), The two-RNP case. Z_NP_ is the position of the center-of-mass of the NP. In order to clearly clarify the changes of force, the corresponding central line of each force profile is highlighted by the bold line with the same color. For comparison, the resistance force in the one-NP case is also shown. $V_{s}=0.004r_{c}/\Delta t$.

In comparison with one-NP case, the resistance forces of NPs in the penetration process change. Here, we take the two-NP case as an example. For the WNPs, their resistance forces abruptly reduce to near zero at the final stage of the translocation, which indicate the successful penetration of the WNPs. However, for the RNPs, their resistance forces still keep fluctuating around a plateau value at the end of the simulations, although in the one-RNP case the resistance force can reduce to zero finally. For the ONPs and JNPs, the changes of resistance forces are similar to one-NP case. Interestingly, it is found that, for all types of NPs, the resistance force acting on each individual NP in the multiple-NP case is smaller than that in the corresponding one-NP case, especially after the NP’s translocation into the membrane.


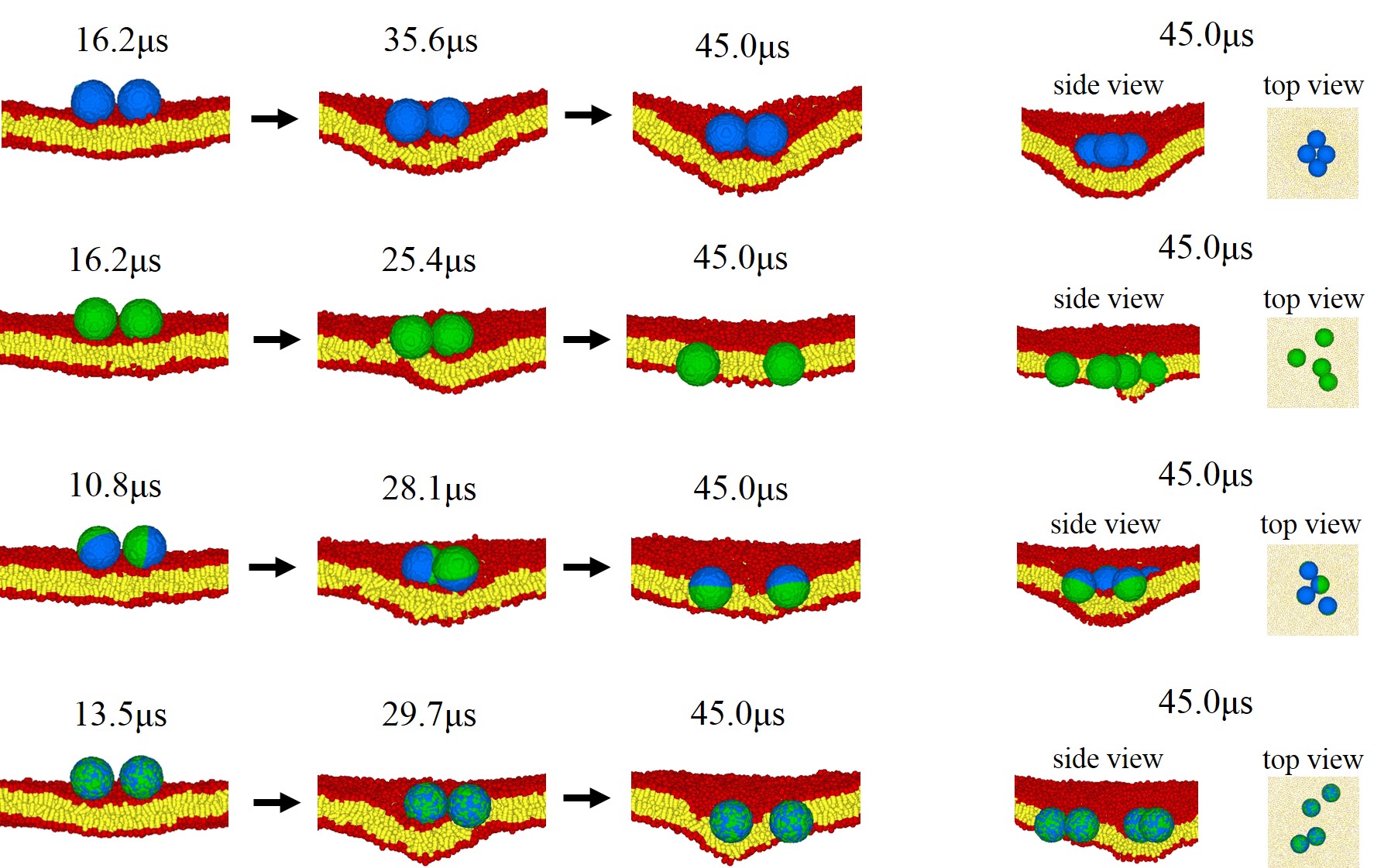


a

b

c

d

e

f

g

h

**Supplementary Fig. S7** Representative translocation process of multiple NPs at . (**a-d**), The two-NP cases. (**e-h**), The four-NP cases. The membranes in the top-view figures are displayed semi-transparently. The initial distance between the nearest-neighbor NPs is $2.4nm$.

The cooperative translocation of NPs is different under a reduced initial driving force with . Under this condition, the WNPs aggregate on the membrane surface and fail to penetrate the membrane due to the weak driving force, while all other types of NPs are trapped in the membrane separately. Comparing the membrane states under these two conditions (i.e., $V_{s}=0.002$ and ), we find that the membrane deformation at $V_{s}=0.002r_{c}/\Delta t$ is gentle. Also, the translocation situations of four NPs are similar to that of the two-NP case. In addition, when $V_{s}=0.004r_{c}/\Delta t$, the four NPs can form regular structures during the translocation process (Fig. 3e-h in the main text). However, when $V_{s}=0.002r_{c}/\Delta t$, except the WNPs, the other NPs disorderly distribute in the membrane at the end of the simulations.

a b


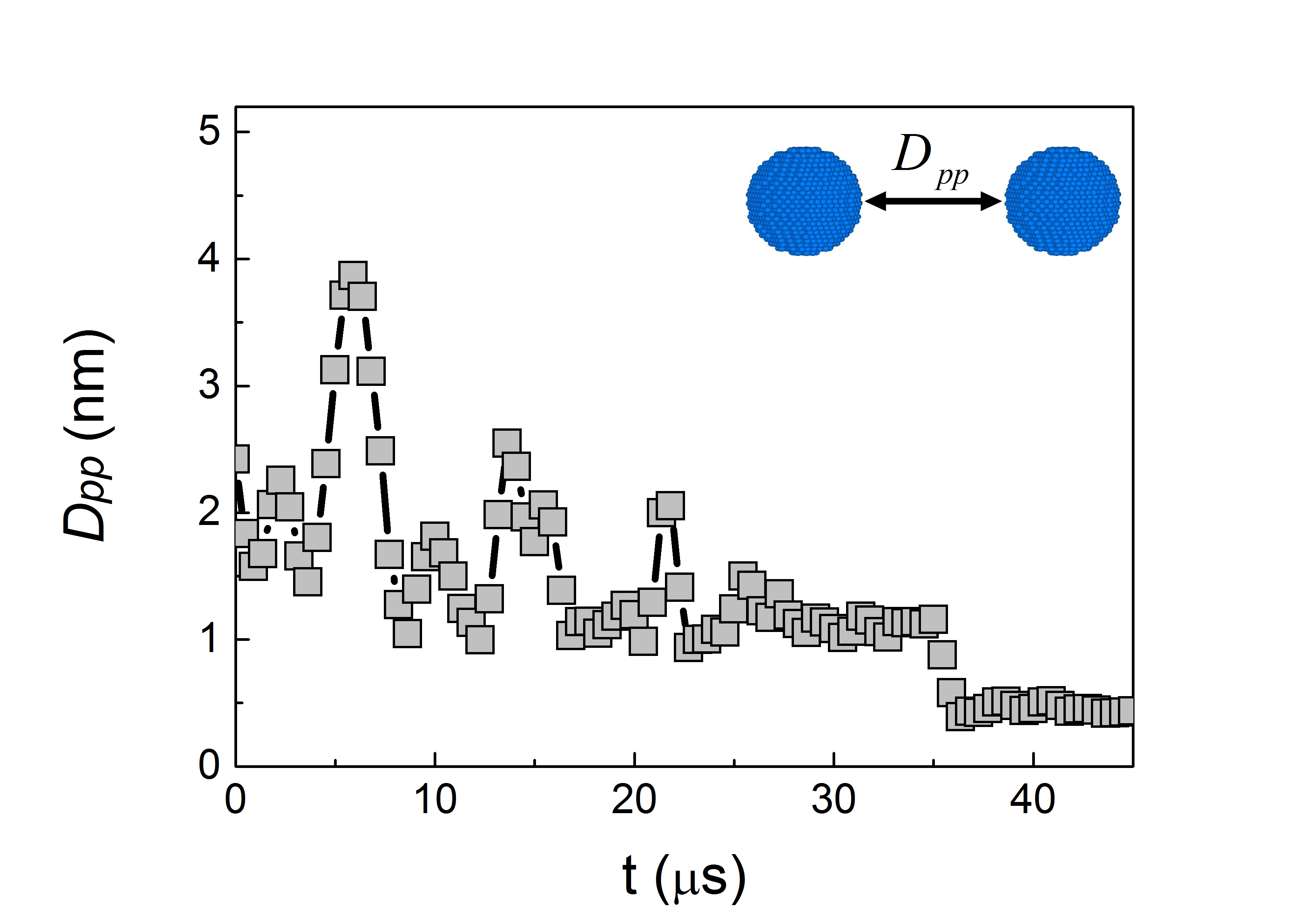

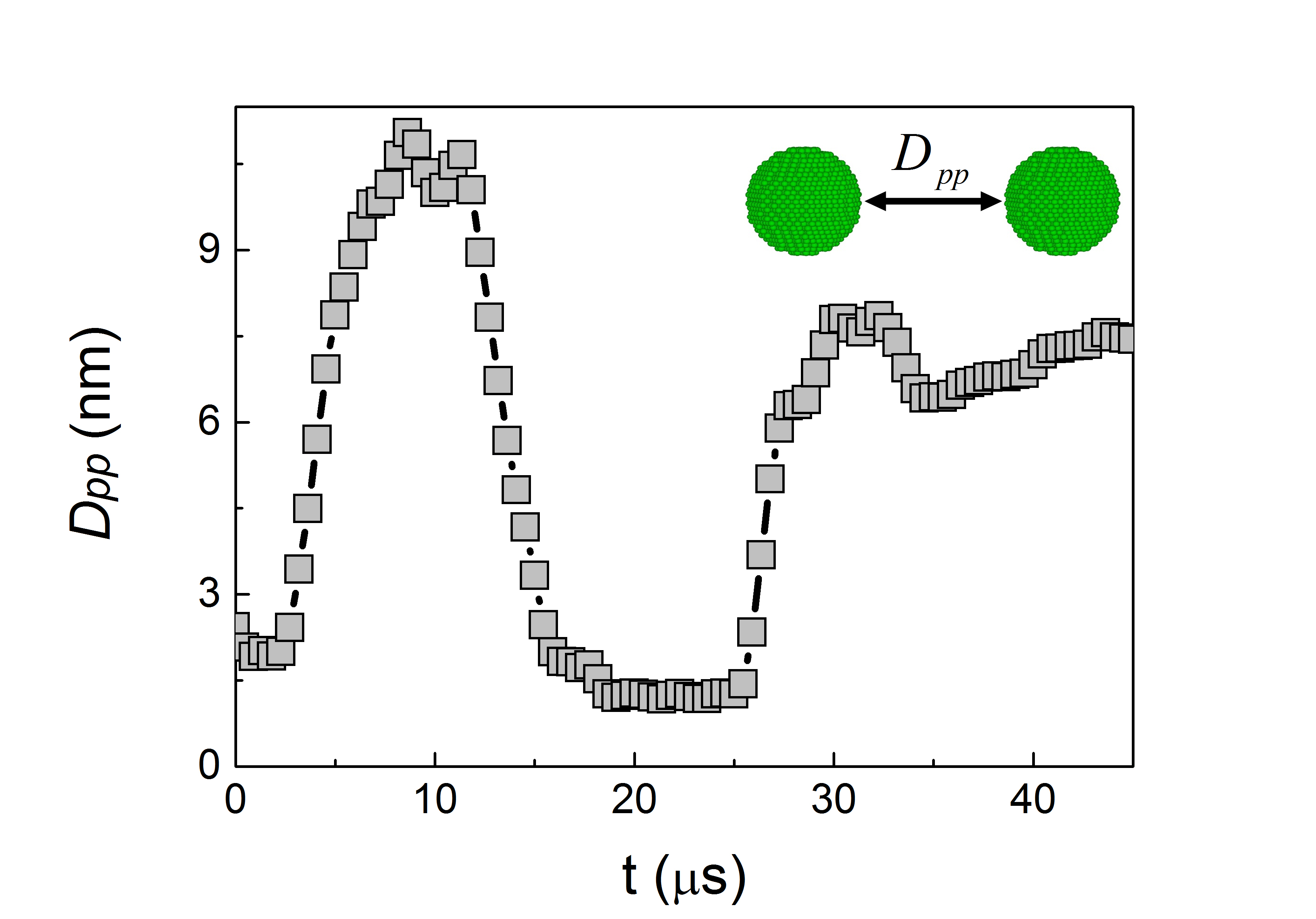


c d


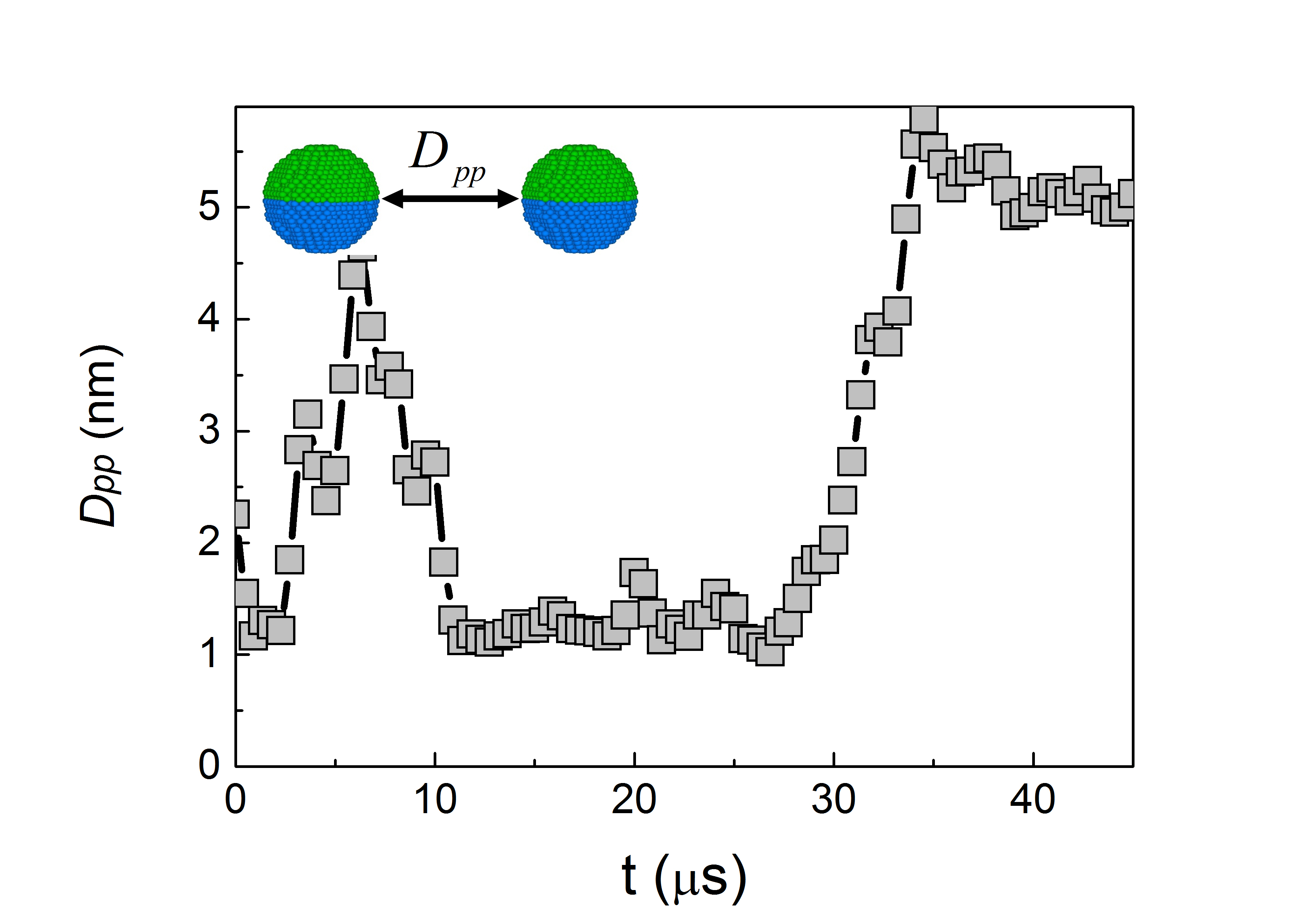

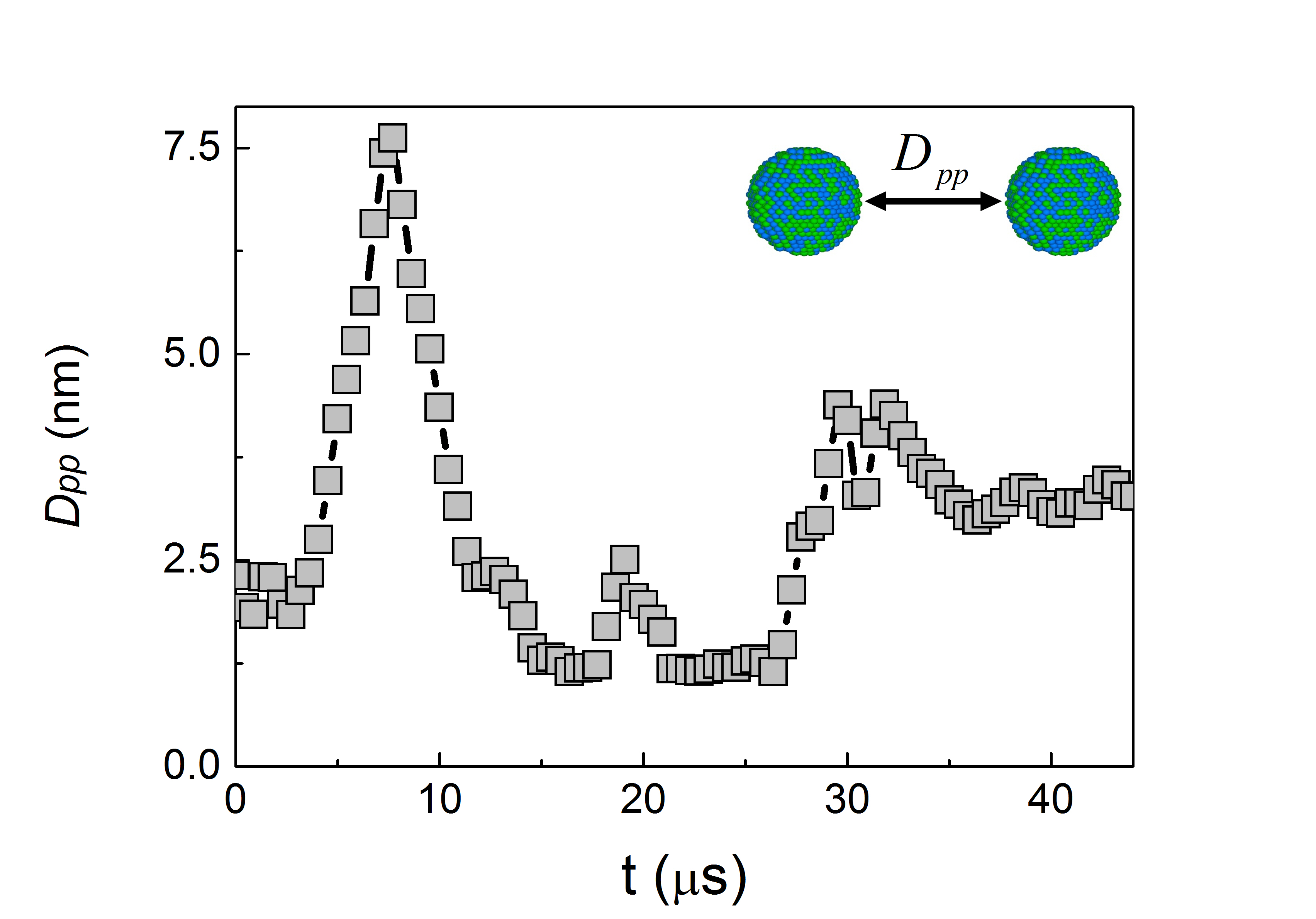


**Supplementary Fig. S8** Typical changes of the distance between the two NPs in the particle penetration process into a membrane. (**a**), WNPs, (**b**), ONPs, (**c**), JNPs, and (**d**), RNPs. The initial distance is $2.4nm$. $V_{s}=0.002r_{c}/\Delta t$.

The inter-NP distance changes of the NPs during the penetration process at $V_{s}=0.002r_{c}/\Delta t$ are shown above. It is found that, for the WNPs, they aggregate in the penetration process. But for other three types of NPs, a rough aggregation-dispersion behavior is observed.

a e


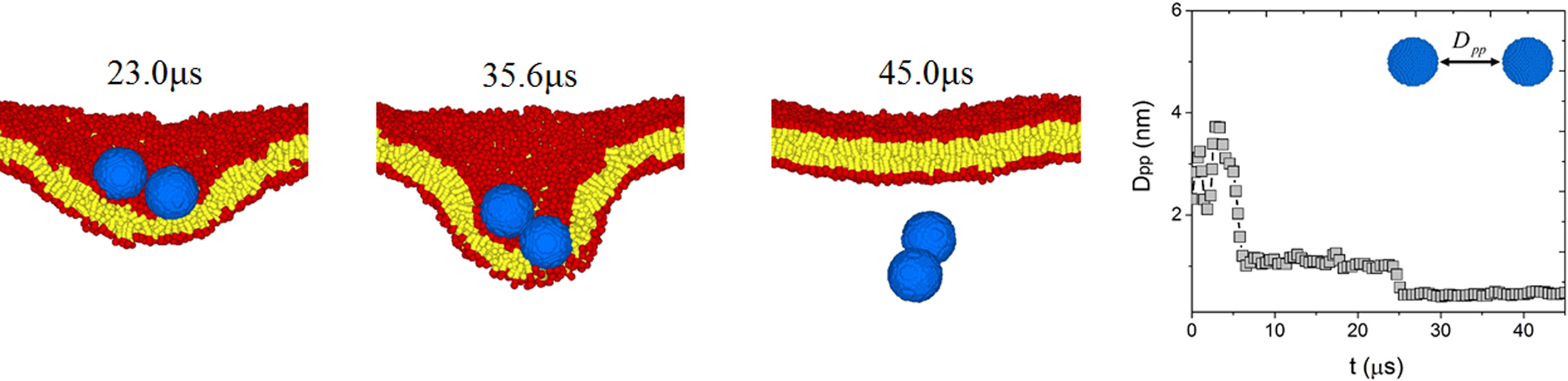


b f


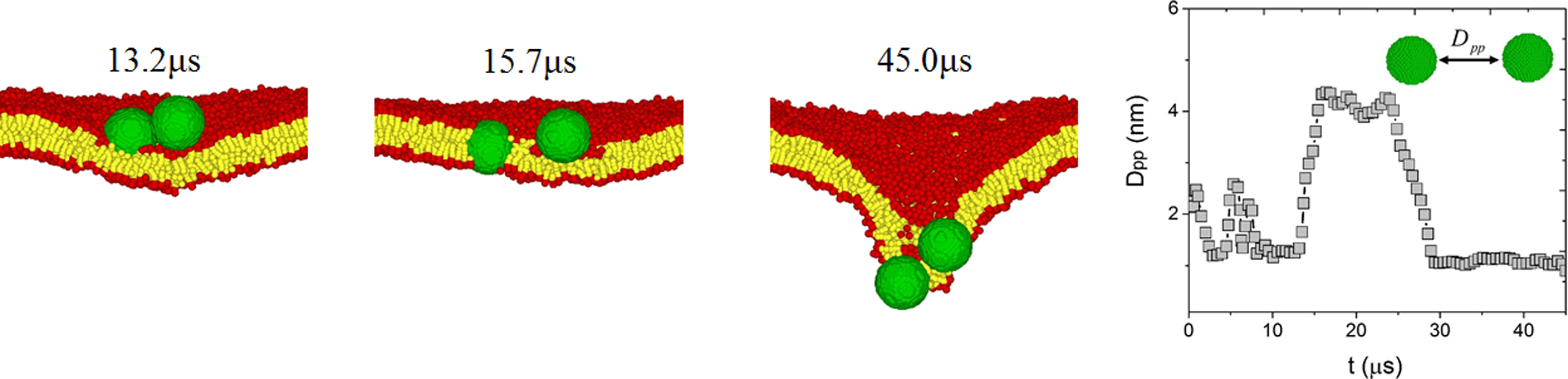


c g


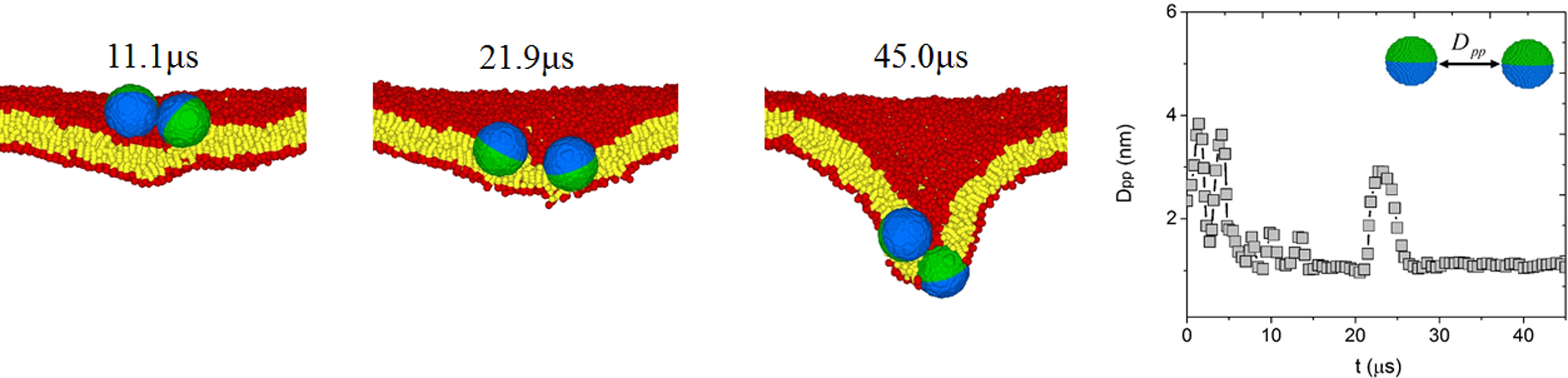


d h


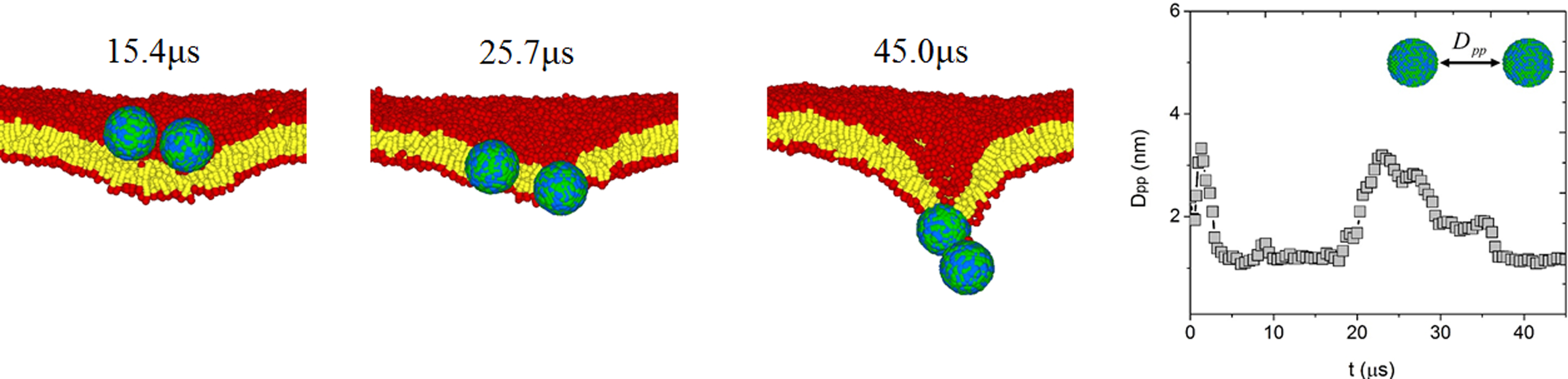


**Supplementary Fig. S9** Representative translocation process of two NPs driven by different velocities ($V_{s}=0.004r_{c}/\Delta t$ and $0.006r_{c}/\Delta t$). (**a**), WNPs. (**b**), ONPs. (**c**), JNPs. (**d**), RNPs. (**e-h**), Corresponding changes of the distance between the two NPs in the penetration process. (**e**), WNPs, (**f**), ONPs, (**g**), JNPs, and (**h**), RNPs. The initial distance is $2.4nm$.

In real experimental or biological environments, the membrane penetration of NPs is possibly guided by different velocities. Therefore, simulations are also carried out to investigate such a situation (one is driven by $V_{s}=0.004r_{c}/\Delta t$ and the other is driven by $V_{s}=0.006r_{c}/\Delta t$). It is found that, in this situation, the NPs always translocate into/through the membrane in a certain order: due to the large driving force, the NP with large $V_{s}$ deforms the membrane quickly and strongly, and thus the other NP with small $V_{s}$tends to follow its penetration (Supplementary Fig. S9). Accordingly, the configurations of NPs and the membrane at the end of the simulations also change: the positions of NPs relative to the membrane are different; but in the case with equal $V_{s}$, the NPs always locate at the similar positions (e.g., Fig. 3 of the text). Furthermore, besides the two-WNP cases, the successful transmembrane penetration of one RNP is also observed due to the large driving force ($V_{s}=0.006r_{c}/\Delta t$). The JNPs and ONPs are still trapped in the membrane at the end of the simulations. However, the cooperative ways of NPs in this situation (with different $V_{s}$) are still similar to the case with equal $V_{s}$: for the WNPs, they aggregate closely in the penetration process, but for the other three groups of NPs (ONPs, JNPs and RNPs), they still adopt an aggregation-dispersion-reaggregation mode (Supplementary Fig. S9).

a b c d


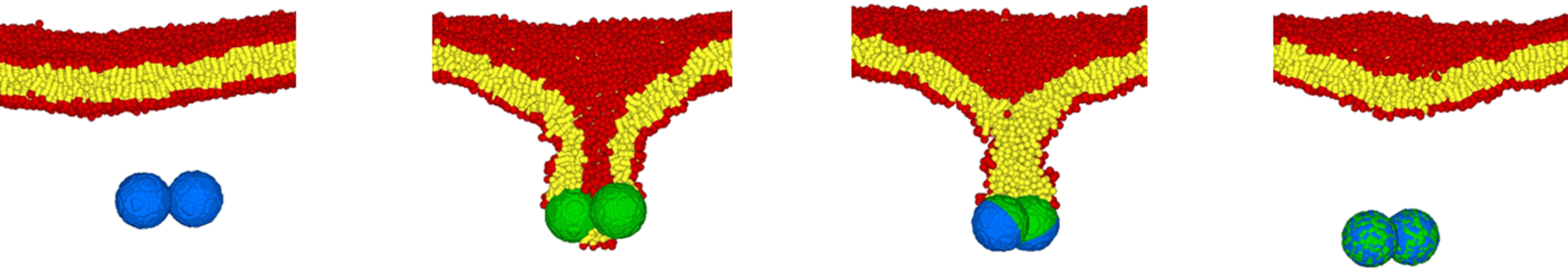


e f g h


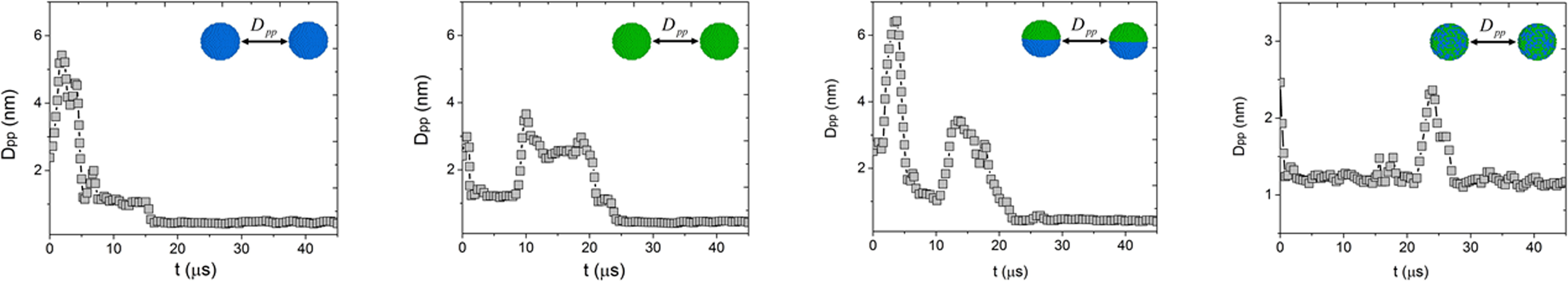


**Supplementary Fig. S10** Representative translocation process of two NPs at $V_{s}=0.006r_{c}/\Delta t$. (**a**), WNPs. (**b**), ONPs. (**c**), JNPs. (**d**), RNPs. (**e-h**), Typical changes of the distance between the two NPs in the penetration process. (**e**), WNPs, (**f**), ONPs, (**g**), JNPs, and (**h**), RNPs. The initial distance is $2.4nm$.

The membrane penetration behaviors of NPs guided by a larger $V_{s}$ ($V_{s}=0.006r_{c}/\Delta t$) are investigated here. With the increase of $V_{s}$, the probability for the NPs to realize the successful membrane penetration increases (e.g., the two-RNP case). But they still follow the similar cooperative modes in the penetration process: the aggregation mode for the WNPs and the aggregation-dispersion-reaggregation mode for ONPs, JNPs and RNPs.

a c e g


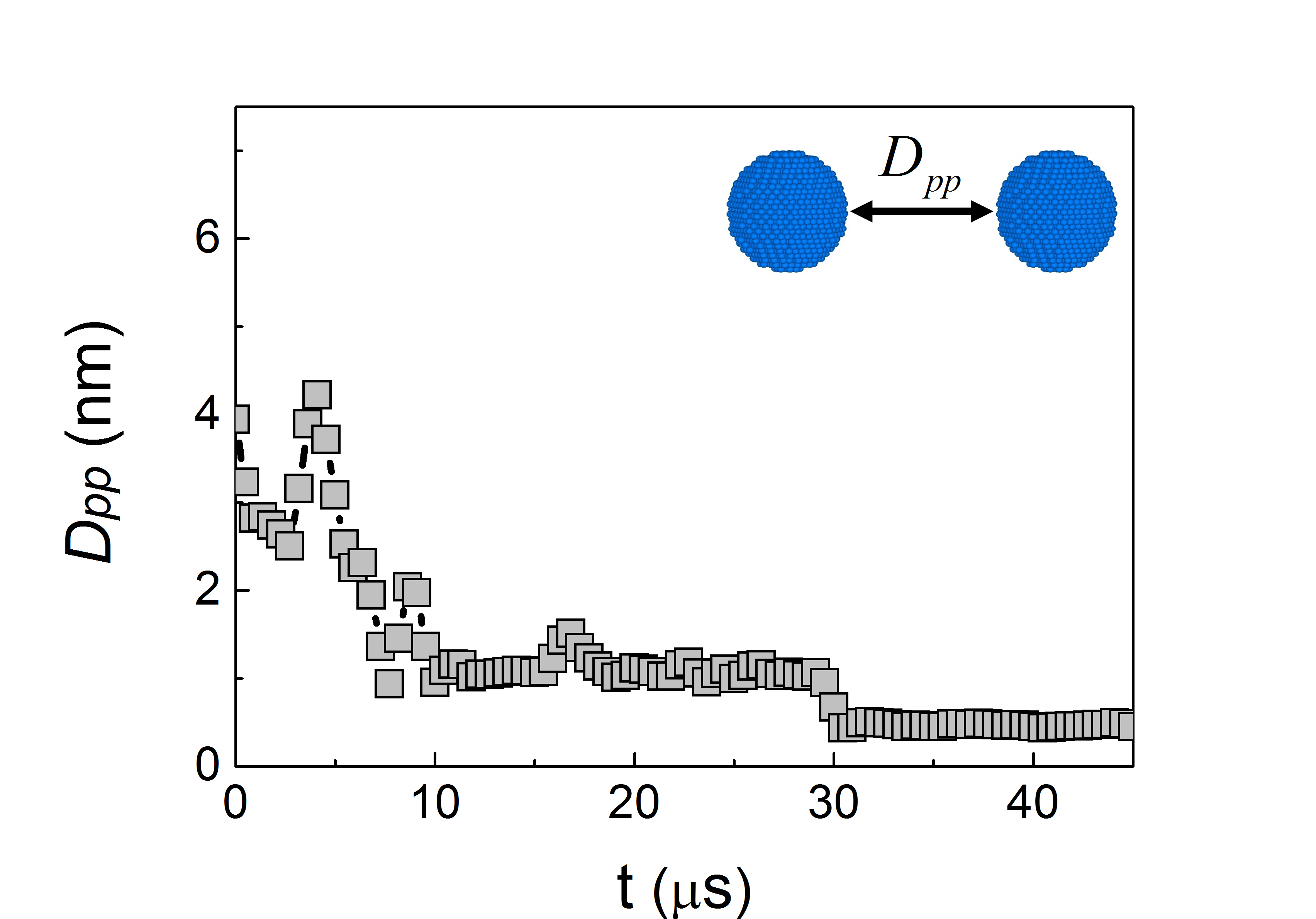

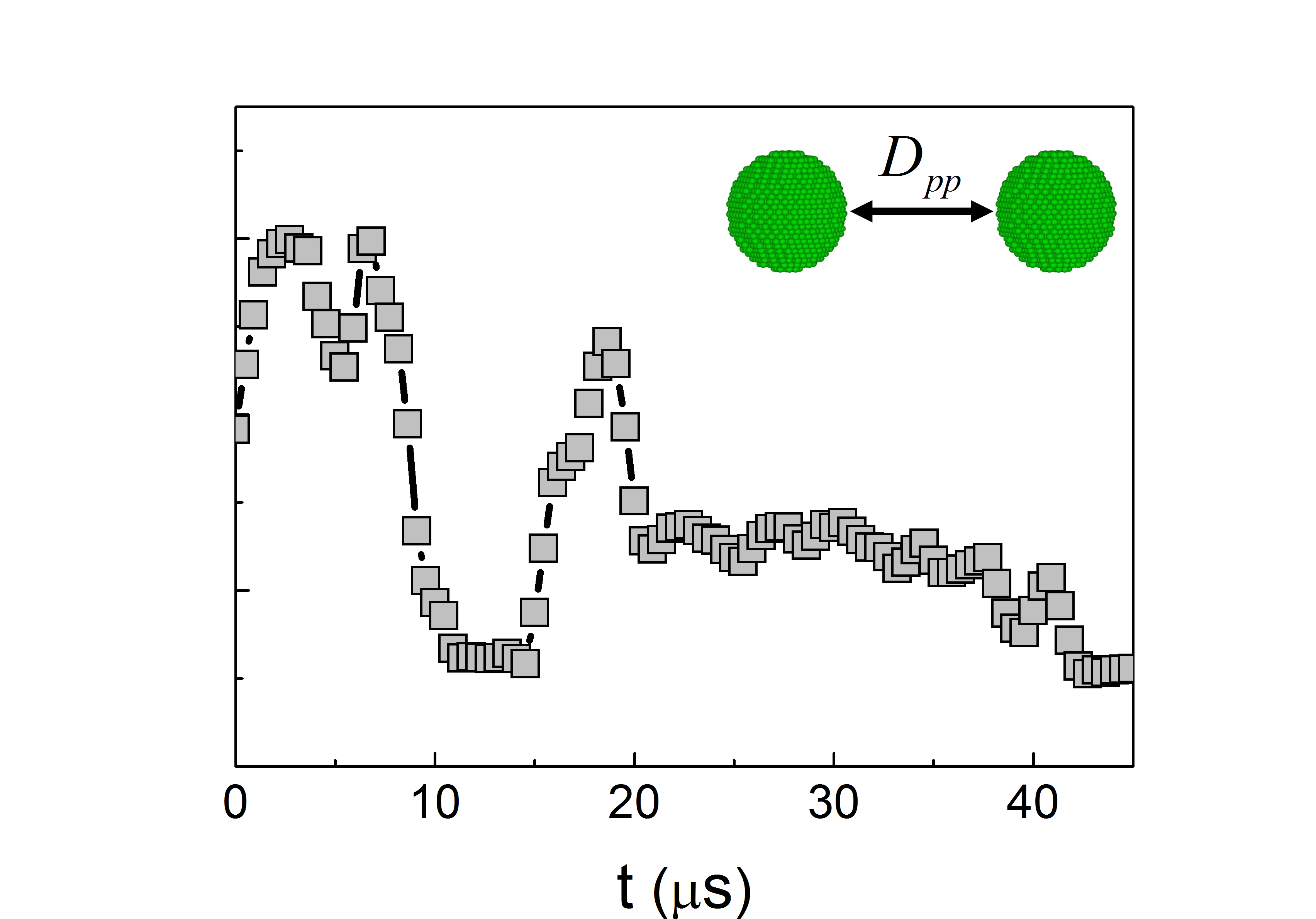

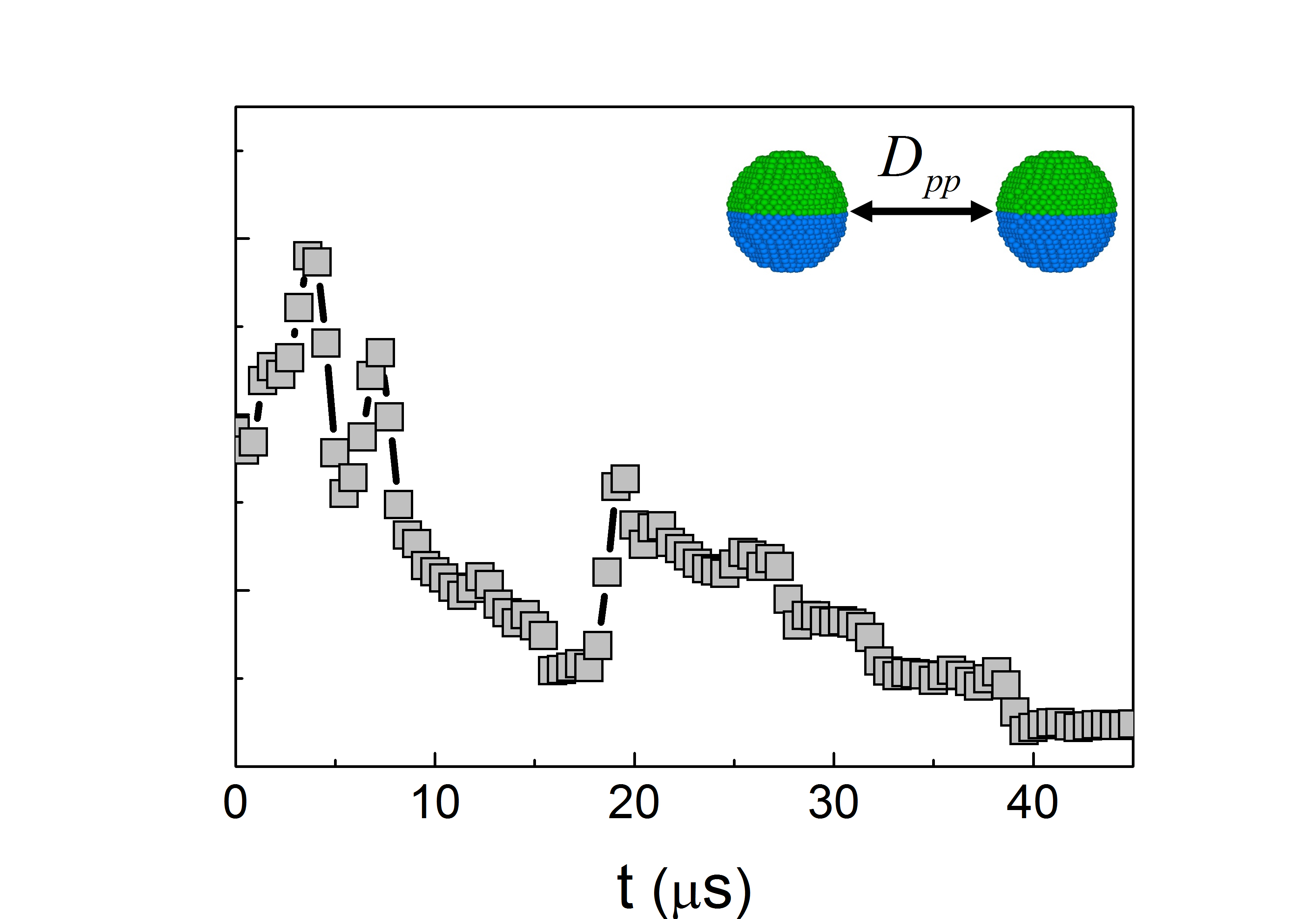

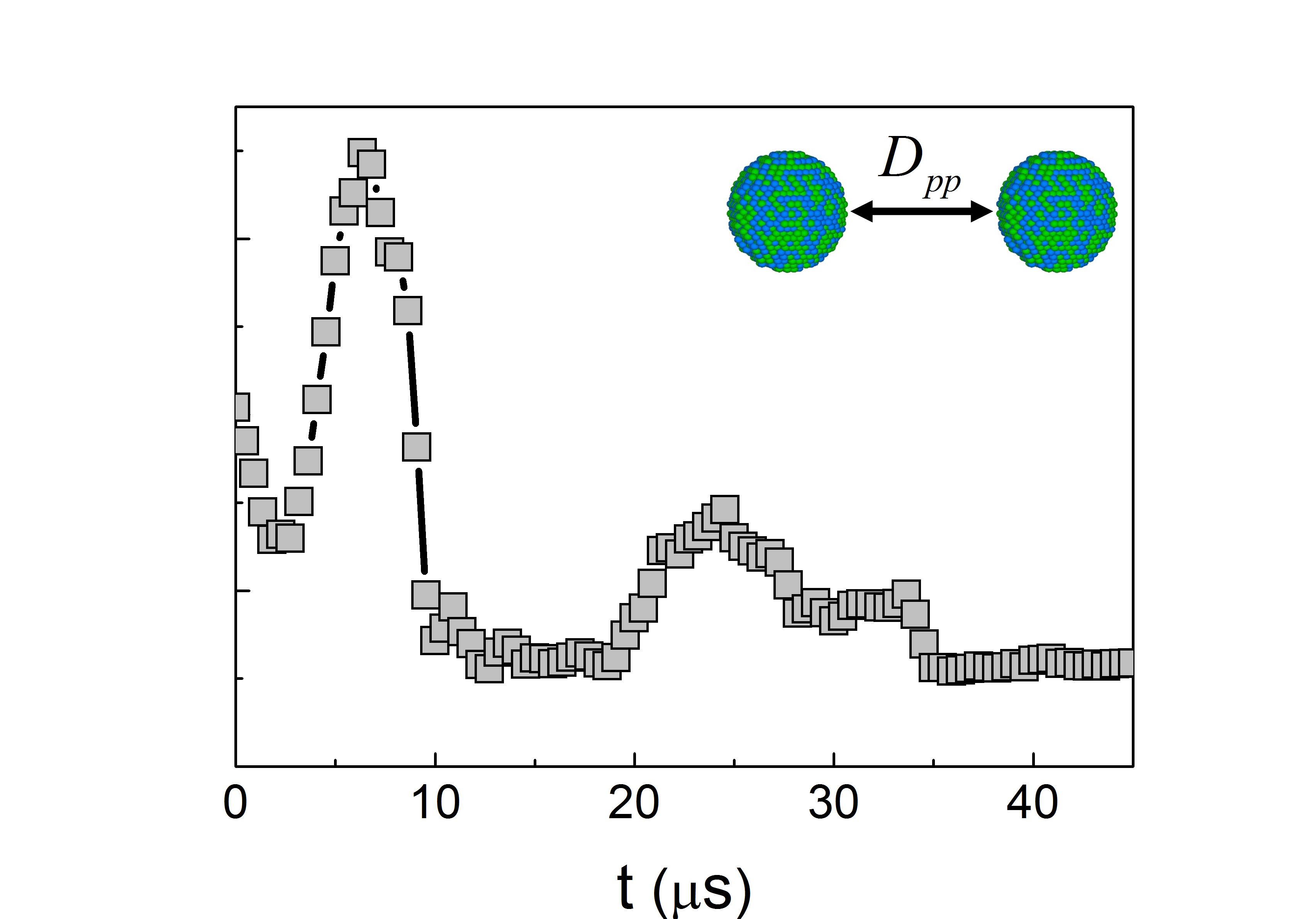


b d f h


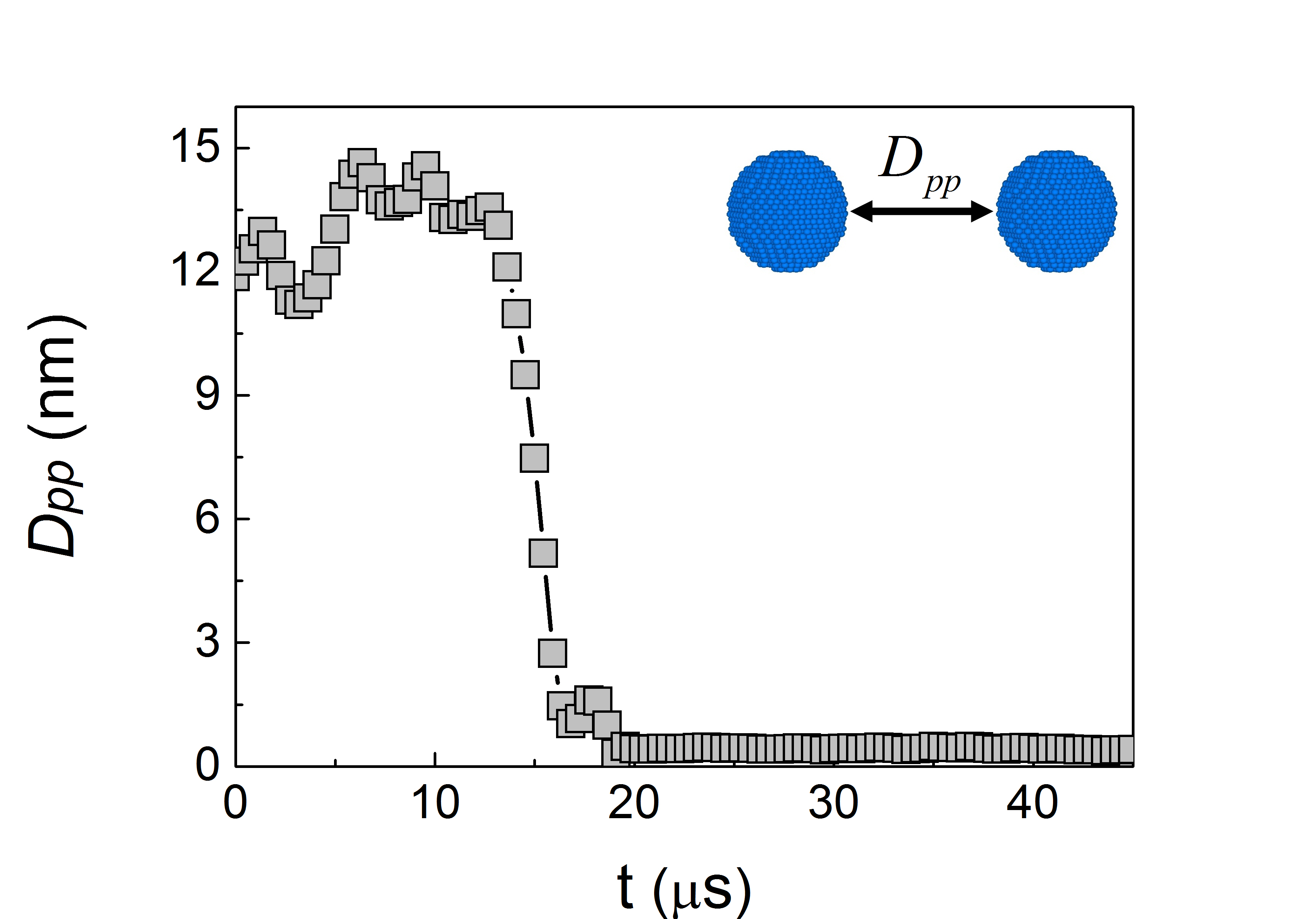

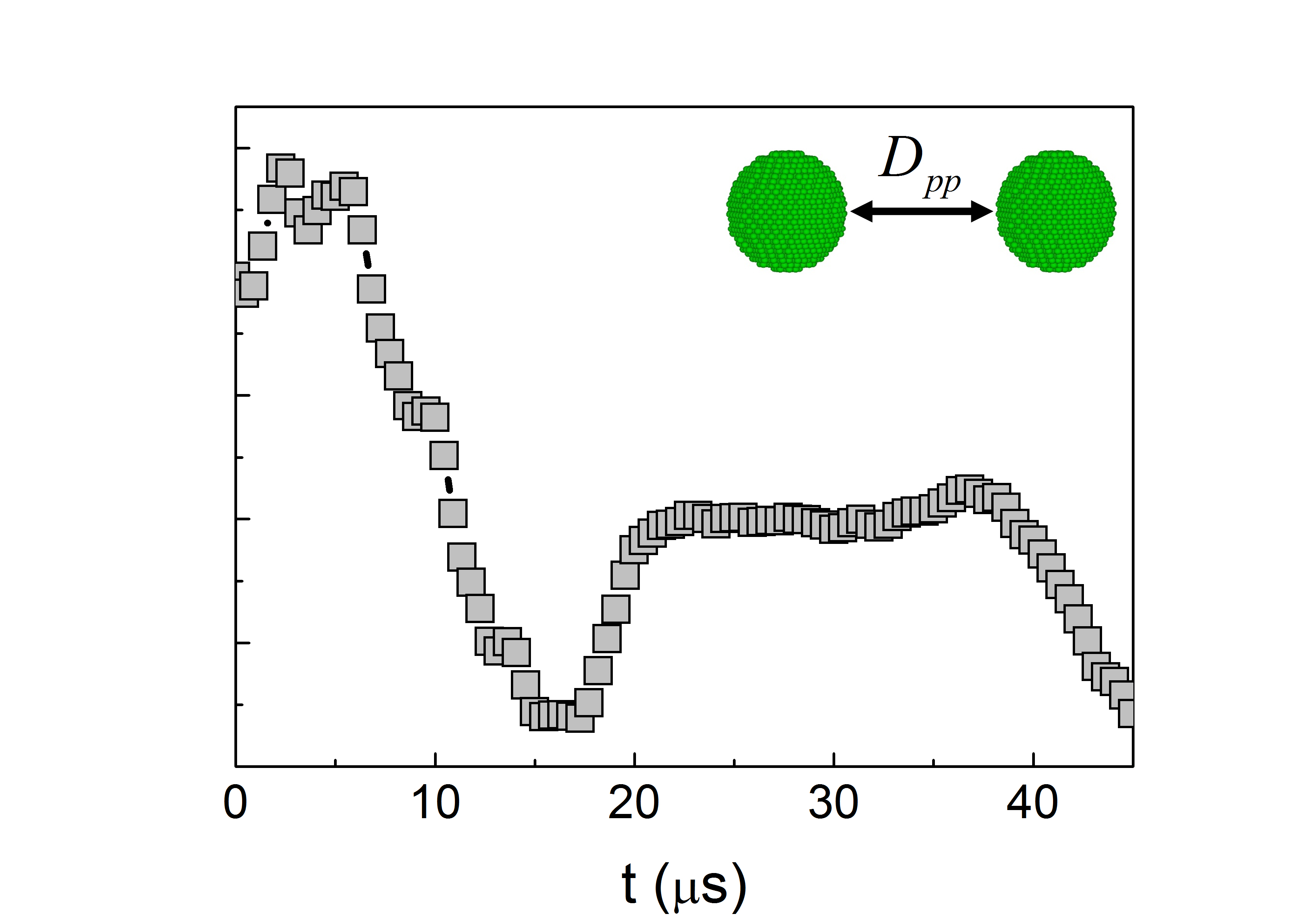

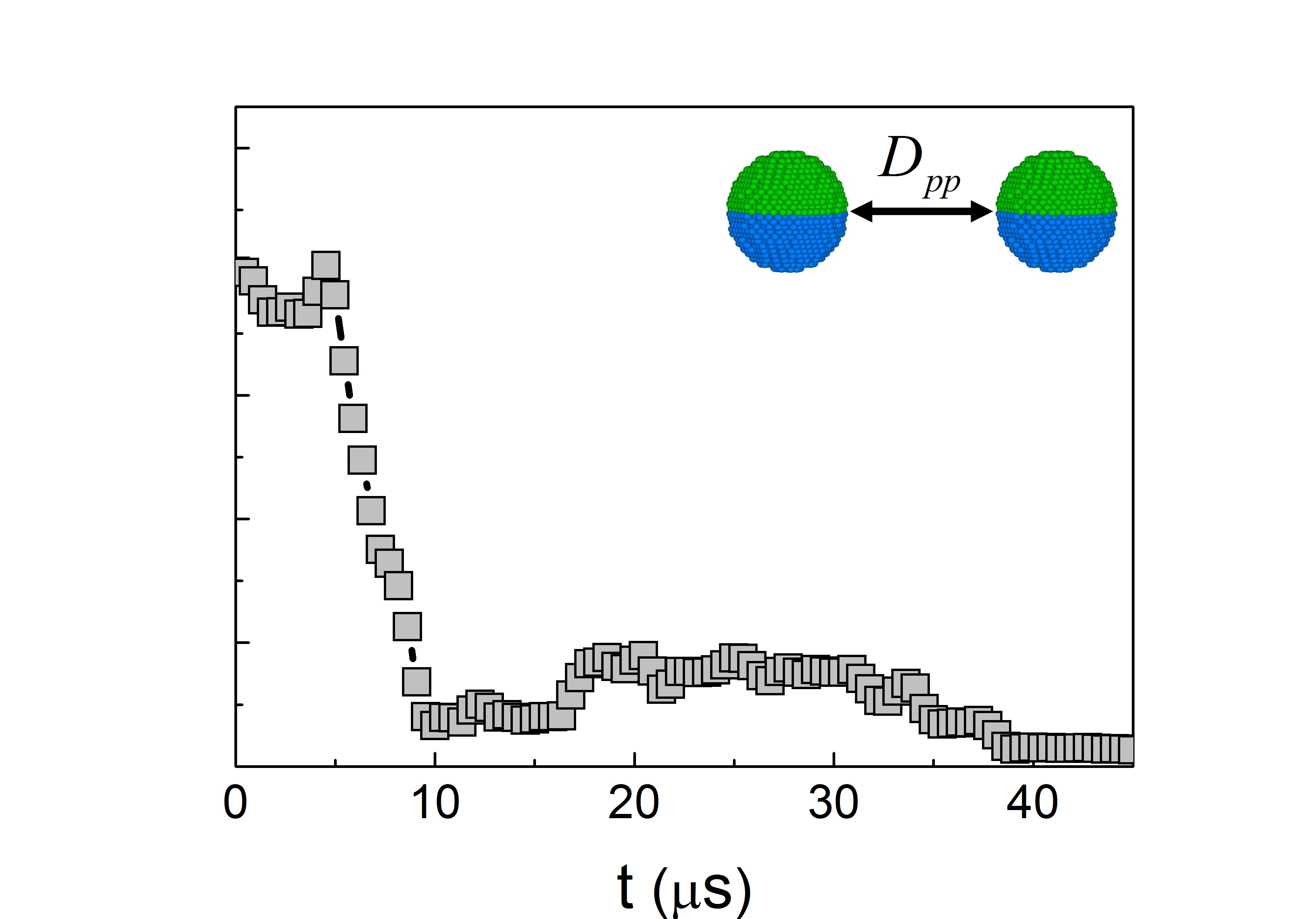

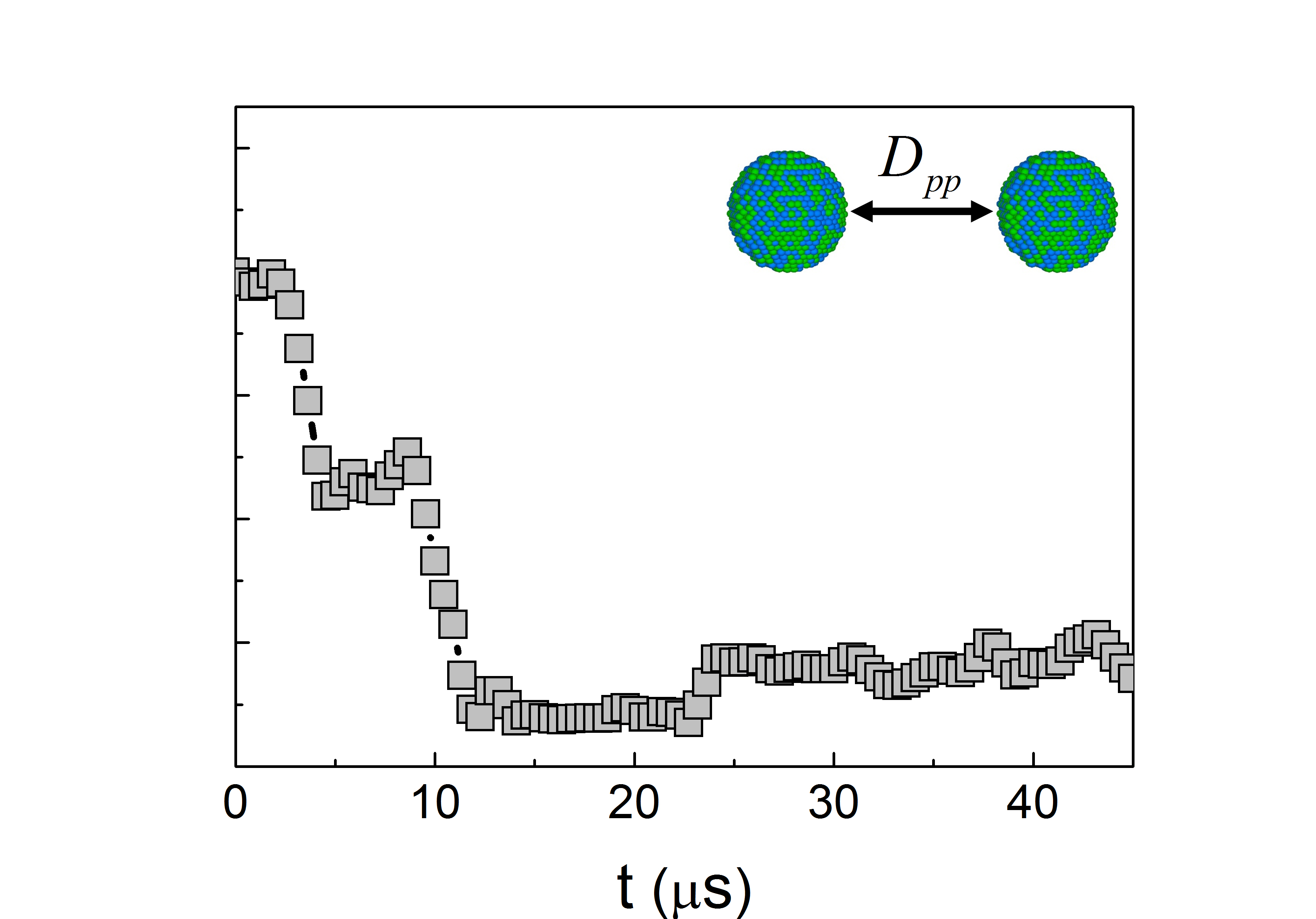


**Supplementary Fig. S11** Typical changes of the distance between two NPs with different initial distances. (**a**, **b**), WNPs; (**c**, **d**), ONPs; (**e**, **f**), JNPs; (**g**, **h**), RNPs. The initial distance is $4nm$ for (**a**, **c**, **e**, **g**) and $12nm$ for (**b**, **d**, **f**, **h**). $V_{s}=0.004r_{c}/\Delta t$.

The influence of initial distance between two NPs is investigated. Here, the results of $D_{pp,t=0}=4nm$ and $12nm$ are shown. It is found that, in the scopes investigated in the simulations, the change of the initial distance between two NPs has a small effect on the cooperative way of the NPs in the membrane plane. Similar to the case of $D_{pp,t=0}=2.4nm$, the aggregation-dispersion-reaggregation of two NPs is observed for the ONPs, JNPs, and RNPs, and the aggregation is found for the WNPs.

**References**

1 Li, Y., Zhang, X. & Cao, D. A spontaneous penetration mechanism of patterned nanoparticles across a biomembrane. *Soft Matter* **10**, 6844-6856 (2014).

2 Liu, J. *et al.* Dewetting transition assisted clearance of (NFGAILS) amyloid fibrils from cell membranes by graphene. *J. Chem. Phys.* **141**, 22D520 (2014).

3 Monticelli, L., Salonen, E., Ke, P. C. & Vattulainen, I. Effects of carbon nanoparticles on lipid membranes: a molecular simulation perspective. *Soft Matter* **5**, 4433-4445 (2009).

4 Li, Y., Li, X., Li, Z. & Gao, H. Surface-structure-regulated penetration of nanoparticles across a cell membrane. *Nanoscale* **4**, 3768-3775 (2012).
